# Clinical significance of *BRAF* non-V600E mutations on the therapeutic effects of anti-EGFR monoclonal antibody treatment in patients with pretreated metastatic colorectal cancer: the Biomarker Research for anti-EGFR monoclonal Antibodies by Comprehensive Cancer genomics (BREAC) study

**DOI:** 10.1038/bjc.2017.308

**Published:** 2017-10-03

**Authors:** Eiji Shinozaki, Takayuki Yoshino, Kentaro Yamazaki, Kei Muro, Kensei Yamaguchi, Tomohiro Nishina, Satoshi Yuki, Kohei Shitara, Hideaki Bando, Sachiyo Mimaki, Chikako Nakai, Koutatsu Matsushima, Yutaka Suzuki, Kiwamu Akagi, Takeharu Yamanaka, Shogo Nomura, Satoshi Fujii, Hiroyasu Esumi, Masaya Sugiyama, Nao Nishida, Masashi Mizokami, Yasuhiro Koh, Yukiko Abe, Atsushi Ohtsu, Katsuya Tsuchihara

**Affiliations:** 1 Department of Gastrointestinal Oncology, Cancer Institute Hospital of Japanese Foundation for Cancer Research, Tokyo 135-0063, Japan; 2 Department of Gastroenterology and Gastrointestinal Oncology, National Cancer Center Hospital East, Kashiwa 277-8577, Japan; 3 Division of Gastrointestinal Oncology, Shizuoka Cancer Center, Shizuoka 411-8777, Japan; 4 Department of Clinical Oncology, Aichi Cancer Center Hospital, Nagoya 464-8681, Japan; 5 Department of Gastroenterology, Saitama Cancer Center, Saitama 362-0806, Japan; 6 Department of Gastrointestinal Medical Oncology, National Hospital Organization Shikoku Cancer Center, Matsuyama 791-0280, Japan; 7 Department of Gastroenterology and Hepatology, Hokkaido University Hospital, Sapporo 060-8648, Japan; 8 Division of Translational Genomics, Exploratory Oncology Research and Clinical Trial Center, National Cancer Center, Chiba 277-8577, Japan; 9 G&G Science Co. Ltd., Fukushima 960-1242, Japan; 10 Department of Computational Biology, Graduate School of Frontier Sciences, The University of Tokyo, Chiba 277-8562, Japan; 11 Division of Molecular Diagnosis and Cancer Prevention, Saitama Cancer Center, Saitama 362-0806, Japan; 12 Department of Biostatistics, National Cancer Center, Kashiwa 277-8577, Japan; 13 Biostatistics Division, Center for Research and Administration and Support, National Cancer Center, Kashiwa 277-8577, Japan; 14 Division of Pathology, Exploratory Oncology Research & Clinical Trial Center, National Cancer Center, Kashiwa 277-8577, Japan; 15 Research Institute for Biomedical Sciences, Tokyo University of Science, Chiba 278-8510, Japan; 16 Genome Medical Science Project, Research Center for Hepatitis and Immunology, National Center for Global Health and Medicine, Chiba 272-8516, Japan; 17 Third Department of Internal Medicine, Wakayama Medical University, Wakayama 641-8509, Japan; 18 Exploratory Oncology Research & Clinical Trial Center, National Cancer Center, Kashiwa, Chiba 277-8577, Japan

**Keywords:** *BRAF*^V600E^ mutation, *BRAF*^non-V600E^ mutation, *RAS* mutation, metastatic colorectal cancer, anti-epidermal growth factor receptor monoclonal antibody

## Abstract

**Background:**

Patients with *BRAF*^V600E^-mutated metastatic colorectal cancer (mCRC) have a poorer prognosis as well as resistance to anti-EGFR antibodies. However, it is unclear whether *BRAF* mutations other than *BRAF*^V600E^ (*BRAF*^non-V600E^ mutations) contribute to anti-EGFR antibody resistance.

**Methods:**

This study was composed of exploratory and inference cohorts. Candidate biomarkers identified by whole exome sequencing from super-responders and nonresponders in the exploratory cohort were validated by targeted resequencing for patients who received anti-EGFR antibody in the inference cohort.

**Results:**

In the exploratory cohort, 31 candidate biomarkers, including *KRAS*/*NRAS*/*BRAF* mutations, were identified. Targeted resequencing of 150 patients in the inference cohort revealed 40 patients with *RAS* (26.7%), 9 patients with *BRAF*^V600E^ (6.0%), and 7 patients with *BRAF*^non-V600E^ mutations (4.7%), respectively. The response rates in *RAS*, *BRAF*^V600E^, and *BRAF*^non-V600E^ were lower than those in *RAS*/*BRAF* wild-type (2.5%, 0%, and 0% *vs* 31.9%). The median PFS in *BRAF*^non-V600E^ mutations was 2.4 months, similar to that in *RAS* or *BRAF*^V600E^ mutations (2.1 and 1.6 months) but significantly worse than that in wild-type *RAS*/*BRAF* (5.9 months).

**Conclusions:**

Although *BRAF*^non-V600E^ mutations identified were a rare and unestablished molecular subtype, certain *BRAF*^non-V600E^ mutations might contribute to a lesser benefit of anti-EGFR monoclonal antibody treatment.

*KRAS* exon 2 mutations were the first validated predictive biomarker for primary resistance to anti-epidermal growth factor receptor (EGFR) monoclonal antibodies (cetuximab and panitumumab) in patients with metastatic colorectal cancer (mCRC) (Amado *et al*, 2008; Van Cutsem *et al*, 2009). Recently, minor *RAS* (*KRAS* exons 3 and 4 or *NRAS* exons 2, 3, and 4) mutations observed in ∼15–20% of mCRC with wild-type *KRAS* exon 2 cases have been validated as negative predictive biomarkers for anti-EGFR antibody treatment (Douillard *et al*, 2013). Therefore, expanded *RAS* (*KRAS* and *NRAS*) testing before administration of anti-EGFR antibody treatment has become essential to maximise the therapeutic benefit in patients with mCRC (Taniguchi *et al*, 2015; Van Cutsem *et al*, 2015; Yoshino *et al*, 2015) However, the response rate (RR) to anti-EGFR monotherapy remains low, at ∼20% in later line, in patients with mCRC having wild-type *RAS*, indicating additional biomarkers beyond expanded *RAS* are needed (Peeters *et al*, 2013).

BRAF is a serine-threonine kinase, located downstream of EGFR in the Ras/Raf/mitogen-activated protein kinase (MAPK) pathway (Mercer and Pritchard, 2003; Roskoski, 2010). The hotspot of *BRAF* mutations in CRC is substitution from valine to glutamic acid at codon 600 (V600E), located in exon 15, leading to 130- to 700-fold increased BRAF kinase activity compared with that of wild-type BRAF; these mutations are reported in ∼5–12% of cases (Davies *et al*, 2002; Andreadi *et al*, 2012). In addition, *BRAF*^V600E^ mutations are more frequently observed in tumours in the right-sided colon than in tumours in the left-sided colon and rectum, and are prognostic biomarkers in CRC and could be potential predictive biomarker for anti-EGFR antibody treatment in pretreated mCRC (Kawazoe *et al*, 2015).

Recent clinical studies have shown that the primary location of the tumour may be associated with the therapeutic effects of anti-EGFR antibody treatment. Tumours in the right-sided colon showed worse outcomes than those in the left-sided colon and rectum in patients with mCRC with wild-type *RAS*, suggesting that genetic alterations other than BRAF V600E could be responsible for the poor prognosis of right-sided tumours (von Einem *et al*, 2014; Brulé *et al*, 2015).

In contrast, few reports have described mCRC with *BRAF* mutations other than *BRAF*^V600E^ (*BRAF*^non-V600E^ mutations), for which the incidence ranges from 1.6% to 5.1% (Shen *et al*, 2013; Ciardiello *et al*, 2014; Cremolini *et al*, 2015). *BRAF*^non-V600E^ mutations can be classified on the basis of kinase activity as either high activity, intermediate activity or impaired activity (130- to 700-fold; high activity mutants, 1.3- to 64-fold; intermediate activity mutants and 30–80%, respectively) (Wan *et al*, 2004). Furthermore, *BRAF* mutation with impaired kinase activity also enhances MAPK kinase (MEK) phosphorylation by heterodimerising with wild-type CRAF (Haling *et al*, 2014). However, little is known regarding the clinicopathological features and anti-EGFR antibody sensitivity of *BRAF*^non-V600E^-mutated mCRC.

Here we reported the clinicopathological features of *BRAF*^non-V600E^-mutated mCRC and the clinical significance of these mutations with regard to the therapeutic effects of anti-EGFR antibody treatment in pretreated mCRC.

## Materials and methods

### Study design

The Biomarker Research for Anti-EGFR Monoclonal Antibodies by Comprehensive Cancer Genomics (BREAC) study was a multicentre, translational research study aiming to investigate novel predictive biomarkers of anti-EGFR antibody treatment in patients with mCRC harbouring wild-type or unknown *KRAS* exon 2 (details in Supplementary Protocol). We had the following study design; patients were divided into two independent cohorts named ‘exploratory’ and ‘inference’ cohorts according to the duration of anti-EGFR antibody treatment. The exploratory cohort included subjects who were considered as ‘super-responders’ or ‘super-nonresponders’ among the entire mCRC cohort (403 patients) who received cetuximab including treatment as salvage line between September 2008 and May 2010 at seven major institutions in Japan. We put a strong assumption that associations between relatively minor gene mutations and patient prognosis become more remarkable in the ‘super-responders’ plus ‘nonresponders’ cohort than associations observed in the entire cohort, leading to a power increase in statistical tests (Supplementary Figure S1). The possible mutations founded in the exploratory cohort were then evaluated by targeted resequencing of the patients in the inference cohort who were treated by anti-EGFR antibody during the different period from the exploratory cohort.

### Study conduct

In the inference cohort, patients with mCRC were consecutively enroled between June 2010 and November 2011 from seven institutions to validate the associations of candidate biomarkers identified in the exploratory cohort with the efficacy of anti-EGFR antibody treatment in pretreated mCRC harbouring wild-type or unknown *KRAS* exon 2. The details of selection criteria for the inference cohort are described in the Supplementary Appendix.

This study was approved by the Institutional Review Board of each participating centre. Written informed consent was obtained from patients who were alive when initiating this study. For deceased patients and their relatives at that time, we disclosed the study design on the website of each centre and allowed the relatives to approve or deny inclusion in the study. This study was conducted in accordance with the Ethical Guidelines for the human genome and genetic analysis research of the Ministry of Education, Culture, Sports, Science and Technology, Ministry of Health, Labour and Welfare and Ministry of Economy, Trade and Industry.

### Collection of clinical and pathological data

An electronic data capture system (Viedoc; PCG Solutions, Uppsala, Sweden) was used for registration of patients and collection of clinical and pathological data by the Office of Translational Research, Exploratory Oncology Research and Clinical Trial Center (EPOC), National Cancer Center, Chiba, Japan.

Patient characteristics including age, sex, site of primary lesion, histology, site of metastases, prior treatments, clinical outcome of anti-EGFR antibody treatment, subsequent treatment, and severe adverse events related to anti-EGFR antibody treatment, were collected. Sites of primary lesions were divided into right-sided colon, left-sided colon, and rectum. Right-sided tumours were defined as those arising anywhere from the caecum to the transverse colon, and left-sided tumours were defined as those arising anywhere from the splenic flexure to the rectosigmoid junction.

Primary investigators were blinded to cancer genome alterations analysed in the study; investigators evaluated the antitumour effect according to Response Evaluation Criteria in Solid Tumours (RECIST) version 1.1 (Eisenhauer *et al*, 2009) and confirmed the safety of the treatment based on the Common Terminology Criteria for Adverse Events (CTCAE) version 4.0 (National Cancer Institute, 2009).

### Targeted capture resequencing

Archived FFPE tissue specimens collected before administration of anti-EGFR antibody were used for target resequencing. Candidate biomarkers identified from the exploratory cohort were validated by target resequencing, which covered the full length of all the candidate genes, including *KRAS*, *NRAS*, and *BRAF*. The details of preparation of clinical samples, DNA extraction, identification of single nucleotide variants (SNVs) and insertion-deletion mutations (INDELs) and target resequencing are described in the Supplementary Appendix.

### BRAF activity assay

To clarify the activity of newly identified *BRAF* mutations, we assessed the phosphorylation status of downstream molecules of EGFR by western blotting using HEK293 cells transfected with the *BRAF* mutant vector (Supplementary Appendix).

### Statistical analysis

The efficacy endpoints were progression-free survival (PFS), defined as the duration from the initiation of anti-EGFR antibody treatment to disease progression or death from any cause; overall survival (OS), defined as the duration from the initiation of anti-EGFR antibody treatment to death from any cause; RR, defined as the proportion of patients who had a complete or partial response with anti-EGFR antibody treatment; and disease control rate (DCR), defined as the proportion of patients who had a complete or partial response or stable disease. For PFS and OS, survival curves according to each mutational status were estimated by the Kaplan–Meier method and were compared using log-rank test.

Univariate and multivariate Cox regression analyses were performed to evaluate the prognostic impact of any *RAS*/*BRAF*^V600E^/*BRAF*^non-V600E^ mutant (herein referred to as *RAS*/*BRAF* mutant) *vs* wild-type. Covariates in the regression analyses included *RAS/BRAF* (mutant *vs* wild-type), age, gender, ECOG PS, histology, primary site, primary tumour resection, adjuvant chemotherapy, metastasis (synchronous *vs* metachronous), combined use of irinotecan, and prior oxaliplatin treatment. Considering the limited number of death events, backward elimination procedure, setting the removal criteria as a *P-*value of <0.20, was performed; four covariates (gender, ECOG PS, primary site, and combined use of irinotecan) were forcibly retained as potential confounding factors.

All statistical analyses were performed using SAS Release 9.3 (SAS Institute, Inc., Cary, NC, USA). All *P* values were obtained from two-sided statistical tests with a significance level of 0.05.

## Results

### Summary of the exploratory cohort

In the exploratory cohort, 92 patients with mCRC, comprising 57 super-responders and 35 nonresponders to anti-EGFR antibody treatment (90 *KRAS* exon 2 wild type and 2 unknown), were selected (Supplementary Figure S2). FFPE clinical samples of both cancerous and noncancerous areas were subjected to whole exon sequencing. Briefly, the exomes were captured using the SureSelect Human All Exon V4+UTRs Kit (Agilent Technologies) and sequenced using a HiSeq 2000 system (Illumina) to generate 100 bppaired-end data. The average base coverage of the targeted regions in the tumour and normal samples was 162.5-fold (range: 10.2–389.7) and 166.2-fold (range: 2.7–377.4), respectively. We identified 182.7±97.1 (range: 37.0–509.0) (5.7±3.0 per Mb, range: 1.2–15.9) somatic SNVs and 8.1±4.2 (range: 1.0–23.0) (0.3±0.1 per Mb, range: 0.0–0.7) somatic INDELs in the tumour tissues. Thirty-one candidate biomarker genes, including *KRAS*/*NRAS*/*BRAF*, in which mutations significantly deviated from either super-responders or nonresponders, were selected for further analysis with the inference cohort. Detailed data of the exploratory cohort is described elsewhere. Here we focused on the association of expanded *RAS* and *BRAF* mutation with efficacy endpoints in the inference cohort.

### Genomic alternations and patient characteristics according to RAS/BRAF status in the inference cohort

A total of 184 patients were selected in the inference cohort. Target resequencing of the candidate biomarker genes, including *KRAS/NRAS* and *BRAF*, was successful in 156 patients, while 28 clinical samples were not analysed due to insufficient FFPE samples (*n*=6) and sequencing failure (*n*=22). The average base coverage of the targeted regions in the tumour and normal samples was 671.9-fold (range: 66.8–1735.0) and 731.6-fold (range: 70.4–1699.5), respectively. We identified 1.4±1.3 (range: 0.0–8.0) variants in the 31 candidate genes from tumour tissues. Additionally, six patients were excluded due to ineligibility (*n*=5) and acquisition of the specimen after anti-EGFR antibody treatment (*n*=1). Accordingly, 150 patients were included in the biomarker analysis population (Supplementary Figure S3). Baseline patient characteristics and clinical outcomes were similar between the whole population (*N*=184) and biomarker analysis population (*N*=150; data not shown).

*KRAS*, *NRAS*, and *BRAF* mutations were detected in 29 (19.3%), 11 (7.3%), and 16 (10.7%) patients, respectively. *RAS* and *BRAF* mutations were identified in a mutually exclusive manner. Nine of 16 *BRAF* mutations (6.0%) were *BRAF*^V600E^ mutations, and seven were *BRAF*^non-V600E^ mutations (4.7%) located in the kinase domain as one G469A (high activated subtype in exon 11) with co-mutation of *MAP2K1*, one L485F (intermediate subtype in exon 12), one Q524L, one L525R (intermediate subtypes in exon 13), two D594G (impaired subtype in exon 15) and one V600R (high subtype in exon 15) with co-mutations of *MSP2* and *PPFIA2* (Table 1). Q524L and L525R were newly identified mutations that were not registered in either the Cancer Genome Atlas (TCGA; http://cancergenome.nih.gov) or the Catalogue of Somatic Mutation in Cancer (COSIMIC; http://cancer.sanger.ac.uk/cosmic) databases.

Baseline patient characteristics, based on the *RAS* and *BRAF* mutational status, are shown in Table 2. Both *BRAF*^V600E^ and *BRAF*^non-V600E^ mutant tumours were more commonly associated with the right-sided colon (44.4% and 57.1%, respectively) than the *RAS*/*BRAF* wild-type and *RAS* mutant tumours (13.8% and 27.5%, respectively). *BRAF*^non-V600E^ mutant tumours tended to have more lymph node metastases (71.4%) than with other mutational subtypes, *RAS* and *BRAF*^V600E^ mutations (27.5% and 11.1%, respectively).

### RRs to anti-EGFR antibody treatment according to RAS/BRAF mutation status in the inference cohort

The RR was 20.7% in all patients. The RRs in patients with *RAS*, *BRAF*^V600E^, and *BRAF*^non-V600E^ mutations were lower in comparison with patients harbouring wild-type *RAS*/*BRAF* (2.5%, 0%, and 0% *vs* 31.9%, respectively). In addition, the proportion of SD more than 6 months in patients with *BRAF*^non-V600E^ mutant was 14.3%, which was similar to that in patients with *RAS* or *BRAF*^V600E^ mutations rather than wild-type *RAS/BRAF* (Table 3).

### Survival and safety analysis according to RAS and BRAF status in the inference cohort

The median follow-up time was 12.1 months as of the cutoff date of December 24, 2014. The median PFS and OS of all patients were 4.0 months (95% confidence interval (CI), 3.4–4.8 months) and 12.4 months (95% CI, 9.8–14.0), respectively.

**Figure 1:**
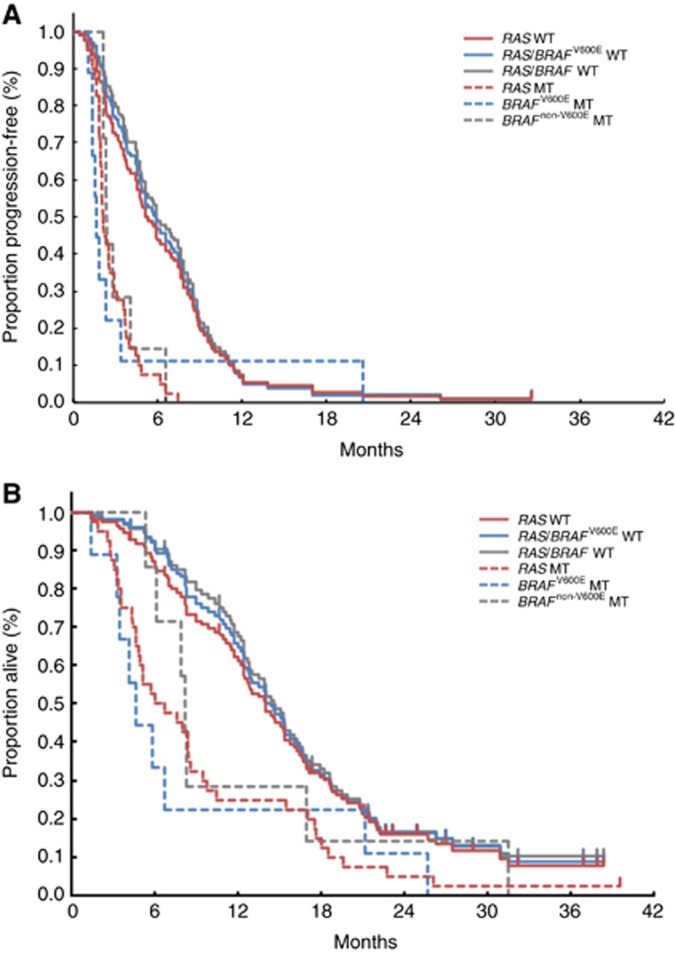
**Merged survival curves for anti-EGFR antibody treatment.** Kaplan–Meier curves for (**A**) progression-free survival (PFS) and (**B**) overall survival (OS) from the initiation of anti-EGFR antibody treatment in patients with pretreated mCRC according to mutational status. Wild-type *RAS*/*BRAF* was defined as all wild-type sequences with *RAS*, *BRAF*^V600E^ and *BRAF*^non-V600E^. A total of 150 patients in the inference cohort were classified according to *RAS*/*BRAF* WT (*n*=94); *RAS* MT (*n*=40); *BRAF*^V600E^ MT (*n*=9); and *BRAF*^non-V600E^ MT (*n*=7). For comparison, Kaplan–Meier curves of *RAS* WT (*n*=110; yellow solid) and *RAS*/*BRAF*^V600E^ WT (*n*=101; blue solid) were added. MT, mutant; WT, wild-type.

The median PFS of patients with *BRAF*^non-V600E^ mutations was 2.4 months (95% CI, 2.1–4.0), similar to that in patients with *RAS* or *BRAF*^V600E^ mutations (2.1 months, 95% CI, 1.9–2.6 and 1.6 months, 95% CI, 1.1–3.4, respectively) but significantly worse than that in patients with wild-type *RAS*/*BRAF* (5.9 months, 95% CI, 4.9–7.7, *P*<0.0001; Table 3, Figure 1).

The median OS of patients with *BRAF*^non-V600E^ mutations was 8.1 months (95% CI, 5.3–16.9), similar to that in patients with *RAS* or *BRAF*^V600E^ mutations (6.3 months, 95% CI, 4.6–8.4 and 4.6 months, 95% CI, 1.3–21.2, respectively) but worse than that in patients with wild-type *RAS*/*BRAF* (14.5 months, 95% CI, 12.6–16.2; Table 3, Figure 1).

### Univariate and multivariate analyses for PFS and OS

Univariate and multivariate analyses for PFS and OS are shown in Table 4. Mutation subtype with *RAS*/*BRAF* was a strong negative prognostic factor for both PFS (HR, 3.49; 95% CI, 2.43–5.00) and OS (HR, 2.14; 95% CI, 1.51–3.04) in univariate analyses. Similarly, the *RAS*/*BRAF* subtype was also a strong negative prognostic factor for both PFS (HR, 5.43; 95% CI, 3.45–8.55) and OS (HR, 3.37; 95% CI, 2.20–5.16) in multivariate analyses.

### BRAF activity assays for the newly identified mutations Q524L and L525R

**Figure 2:**
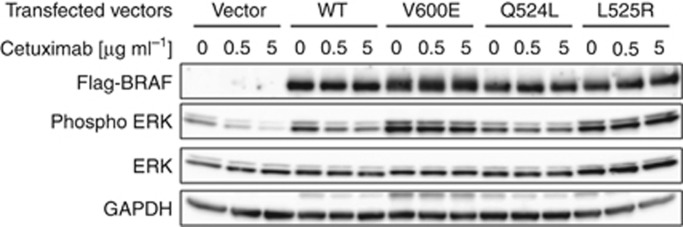
**BRAF activity assay.** Phosphorylation status of ERK was assessed by western blotting. HEK293 cells were transiently transfected with wild-type, V600E, Q524L, or L525R BRAF and were then treated with 0.5 or 5 μg/ml cetuximab.

To evaluate the kinase activity of newly identified BRAF mutants, mutant- and wild-type BRAF-expressing vectors were transiently transfected into EGFR-expressing HEK293 cells. The transfection efficiency was more than 70%, as assessed by EGFR-expressing control plasmid vector transfection (data not shown). Western blot analysis showed that extracellular signal-regulated kinase (ERK) phosphorylation in cells with BRAF^V600E^ overexpression was significantly increased compared with that in cells with wild-type BRAF overexpression (Figure 2). BRAF^L525R^ induced increased ERK phosphorylation to a level similar to that induced by BRAF^V600E^. However, BRAF^Q524L^ activity was similar to that of wild-type BRAF.

In the control vector-transfected cells, cetuximab reduced ERK phosphorylation in a concentration-dependent manner. Additionally, cetuximab reduced ERK phosphorylation level, which was enhanced by wild-type BRAF or BRAF^Q524L^ expression. On the other hand, ERK phosphorylation enhanced by BRAF^V600E^ and BRAF^L525R^ was not affected by cetuximab, suggesting that cells with BRAF^V600E^ or BRAF^L525R^ mutants were resistant to cetuximab-induced inhibition of EGFR.

## Discussion

To the best of our knowledge, this is the first report for the clinical significance of *BRAF*^non-V600E^ mutations focusing on the therapeutic effects of anti-EGFR monoclonal antibodies in patients with pretreated mCRC.

Few studies have reported the clinicopathological features of *BRAF*^non-V600E^ mutations because *BRAF*^V600E^-mutant tumours are most frequently observed in CRC (Shen *et al*, 2013; Ciardiello *et al*, 2014). According to studies in Western countries, the incidence of *BRAF*^non-V600E^ in mCRC was reported to range from 1.6% to 2.7% (Ciardiello *et al*, 2014; Cremolini *et al*, 2015). In contrast, the incidence of *BRAF*^non-V600E^ in 676 Chinese patients with mCRC was reported to be 5.1%, consistent with that in our cohort (4.7%) (Shen *et al*, 2013). On the other hand, the racial differences in terms of the incidence of *BRAF* ^V600E^ mutations were reported from the analysis of large-scale adjuvant trial in US, suggesting the incidence appeared to be lower in Asians than in blacks or whites (Yoon *et al*, 2015). Instead, the incidence of *BRAF*^non-V600E^ mutations might be higher in Asian than that in Caucasian patients.

Two meta-analyses suggested that primary tumours in the right-sided colon showed worse prognoses than those in the left-sided colon and rectum in patients treated with anti-EGFR antibodies (Arnold *et al*, 2017; Holch *et al*, 2017). In addition, integrated analysis of two randomised panitumumab studies showed that they had consistent results, even when the *BRAF*^V600E^ mutations were excluded (Boeckx *et al*, 2017). In our series, a similar tendency was observed clinical outcomes; specifically, patients with wild-type *RAS* mCRC with primary tumours in the right-sided colon had poorer prognoses compared with those having primary tumours in the left-sided colon and rectum, although the difference was not significant. However, if limited to wild-type *RAS/BRAF* tumours, there were no clear differences in OS among sites of primary lesions (Supplementary Figure 4). Thus, the unresponsiveness of primary tumours in the right-sided colon to anti-EGFR antibodies in later line might be partially explained by underlying *BRAF*^non-V600E^-mutated tumours.

Subtypes of *BRAF* mutations in the kinase domain can be classified into high, intermediate, and impaired activity subtypes based on their kinase activity (Wan *et al*, 2004). The *BRAF*^V600E^ mutation belongs to the high activity subtype, whereas the *BRAF*^G469A^, *BRAF*^L485F^, and *BRAF*^V600R^ mutations observed in this study belong to the intermediate subtype and the *BRAF*^D594G^ mutation belongs to the impaired subtype. Moreover, the BRAF^L525R^ mutant observed in this study had enhanced kinase activity, and the enhanced downstream signal of BRAF^L525R^ may contribute to primary resistance to cetuximab, consistent with the lack of response to anti-EGFR antibody treatment in our series. In contrast, the newly identified BRAF^Q524L^ mutant identified in this study had intermediate kinase activity and did not induce resistance to cetuximab in an *in vitro* cell model. However, such *in vitro* experiments in non-CRC epithelial cell lines may not fully predict the clinical outcome.

One possible explanation for the similar behaviours of *BRAF*^non-V600E^ and *BRAF*^V600E^-mutated tumours in terms of unresponsiveness to anti-EGFR antibody treatment may be the incomplete blockade of the MEK pathway by modestly upregulated kinase activity of BRAF and/or by additional signalling through wild-type CRAF (Wan *et al*, 2004). Therefore, it is necessary to establish patient-derived xenograft models harbouring *BRAF*^non-V600E^ mutations to clarify the mechanisms of primary resistance to anti-EGFR antibody treatment in *BRA*F^non-V600E^-mutated mCRC.

Regarding *BRAF*^D594G^, classified as an impaired subtype in the study, Cremolini *et al* reported 10 cases with *BRAF* mutations in codons 594 or 596, the number of which was similarly small showing a favourable prognosis from first line (Cremolini *et al*, 2015). More recently, it was reported that OS was significantly longer for 101 patients with *BRAF*^non-V600E^ mutations than for the control group of 99 patients with *BRAF*^V600E^ mutations (60.7 *vs* 11.4 months) (Jones *et al*, 2017); however, our analysis focused on pretreated population. In addition, according to the European Consortium, De Roock *et al* reported that two patients harbouring *BRAF*^D594G^-mutated mCRC achieved a partial response to cetuximab monotherapy (De Roock *et al*, 2010). In contrast, the two patients with *BRAF*^D594G^-mutated tumours in our study did not achieve objective response to anti-EGFR antibody treatment. Considering small patient’s number of each reports as well as heterogeneous population, it is difficult to conclude predictive impact of *BRAF*^non-V600E^ mutation. The overall data in this study supported that *BRAF*^non-V600E^ mutations were prognostic, as a similar magnitude to the presence of *BRAF*^V600E^ and *RAS* mutations in later line, and the outcome appeared similar to patients with *RAS* mutations as well, who do not benefit from anti-EGFR therapy. The present study has some limitations. It was a retrospective study with a small number of subgroups of *BRAF*^non-V600E^ mutations, using archived FFPE samples. In addition, it is difficult to conduct further analyses by subdividing the group into ‘non-V600E kinase activity’ and ‘non-V600E non- kinase activity’ subgroups, due to the small number of *BRAF*^non-V600E^ mutations. The emergence of secondary *RAS* and *BRAF* gene mutations in ctDNA was recently reported after treatment with anti-EGFR antibody (Bettegowda *et al*, 2014). However, in the case of patients receiving systemic chemotherapy without anti-EGFR antibody, secondary gene alterations are rare (Kawamoto *et al*, 2012); therefore, we believed that adding the archived FFPE samples before anti-EGFR antibody administration would be reasonable. Further investigation in large-scale data set from such as randomised control trials is necessary to clarify the significance of anti-EGFR antibody treatment for each *BRA*F^non-V600E^ mutational variants as the role of the predictive value.

In conclusion, although identified *BRAF*^non-V600E^ mutations were rare and unestablished molecular subtype in mCRC, overall clinical outcomes of *BRAF*^non-V600E^ mutations in the kinase domain, similar to those of *RAS*- and *BRAF*^V600E^-mutant tumours, appeared to be significantly worse than those in wild-type *RAS*/*BRAF* tumours. Certain *BRAF*^non-V600E^ mutations might contribute to a lesser benefit of anti-EGFR monoclonal antibody treatment. This novel knowledge provides an intriguing background to investigate new target approaches in patients with *BRAF*^non-V600E^ mutations and represents substantial progression toward more precision medicine.

## Figures and Tables

**Table 1:** Genomic alternations detected by targeted resequencing of the BRAF^non-V600E^ mutation

**ID**	**GQ0XS**	**GLCH7**	**SC12PCQ3IA02**	**G9OJR**	**GQ4U5**	**GUZG7**	**GS3A5**
hg19 position	140481402	140477853	140476835	140476832	140453154	140453154	140453136-
							140453137
Amino acid variation	G469A	L485F	Q524L	L525R	D594G	D594G	V600R
Kinase activity	Activated	Activated	NR	NR	Impaired	Impaired	Activated
Co-mutations	MAP2K1	—	—	—	—	—	MAP2, PPFIA2

Abbreviation: NR = not reported.

**Table 2:** Baseline patient characteristics according to RAS/BRAF mutational status

	***RAS*****/*****BRAF*** **wild**		***RAS*** **mut**		***BRAF***^**V600E**^ **mut**		***BRAF***^**non-V600E**^ **mut**	
	(***n*****=94)**		(***n*****=40)**		(***n*****=9)**		(***n*****=7)**	
	**No**	**%**	**No**	**%**	**No**	**%**	**No**	**%**
**Age, years**								
Median	64		63		64		63	
Range	28–85		35–79		33–73		48–74	
**Gender**								
Male	64	68.1	18	45	4	44.4	1	14.3
Female	30	31.9	22	55	5	55.6	6	85.7
**ECOG PS**								
0	53	56.4	22	55	3	33.3	3	42.9
1	38	40.4	17	42.5	6	66.7	4	57.1
2	3	3.2	1	2.5	0	0	0	0
**Histology**^a^								
Well	17	18.1	11	27.5	1	11.1	1	14.3
Moderate	62	66	23	57.5	6	66.7	5	71.4
Poor	11	11.7	5	12.5	1	11.1	1	14.3
Others	4	4.3	1	2.5	1	11.1	0	0
**Primary tumour site**^b^								
Right-sided colon	13	13.8	11	27.5	4	44.4	4	57.1
Left-sided colon or rectum	81	86.2	29	72.5	5	55.6	3	42.9
**Resection of primary tumour**								
	77	81.9	35	87.5	8	88.9	7	100
**Time to metastases**								
Synchronous	50	53.2	20	50	4	44.4	1	14.3
Metachronous	44	46.8	20	50	5	55.6	6	85.7
**Number of metastases**								
1	33	35.1	10	25	5	55.6	0	0
>1	61	64.9	30	75	4	44.4	7	100
**Metastatic site**								
Liver	61	64.9	25	62.5	4	44.4	4	57.1
Lung	49	52.1	27	67.5	3	33.3	6	85.7
Peritoneum	20	21.3	9	22.5	3	33.3	2	28.6
Lymph node	37	39.4	11	27.5	1	11.1	5	71.4
**Reason of discontinuation for each cytotoxic agent as prior treatment**								
Fluoropyrimidine								
Failure	94	100	40	100	9	100	7	100
Intolerance	0	0	0	0	0	0	0	0
Oxaliplatin								
Failure	83	88.3	38	95	9	100	5	71.4
Intolerance	11	11.7	2	5	0	0	2	28.6
Irinotecan								
Failure	94	100	40	100	9	100	7	100
Intolerance	0	0	0	0	0	0	0	0
**Prior Bevacizumab**								
	74	78.7	33	82.5	8	88.9	7	100
**Anti-EGFR antibody treatment**								
Cetuximab	66	70.2	29	72.5	6	66.7	5	71.4
Panitumumab	28	29.8	11	27.5	3	33.3	2	28.6
**Combine use of Irinotecan**								
Yes	69	73.4	35	87.5	8	88.9	4	57.1
No	25	26.6	5	12.5	1	11.1	3	42.9

Abbreviations: ECOG=Eastern Cooperative Oncology Group; FP=fluoropyrimidine; mut=mutant; poor=poorly differentiated; well=well differentiated; moderate, moderately differentiated; wild=wild-type.

Wild-type *RAS/BRAF* was defined as all wild-type sequence with *RAS, BRAF*^V600E^, and *BRAF*^non-V600E^.

a Defined according to Japanese Classification (ref: JSCCR, Japanese Classification of Colorectal Carcinoma, 2nd English Ed).

b Right-sided tumours were defined as those arising anywhere from the caecum to the transverse colon, and left-sided tumours were defined as those arising anywhere from the splenic flexure to the rectosigmoid junction.

**Table 3:** Efficacy of anti-EGFR antibody treatment according to the RAS/BRAF mutational status

	***RAS*****/*****BRAF*** **wild**		***RAS*** **mut**		***BRAF***^**V600E**^ **mut**		***BRAF***^**non-V600E**^ **mut**	
	(***n*****=94)**		(***n*****=40)**		(***n*****=9)**		(***n*****=7)**	
**PFS, months**								
Median	5.9		2.1		1.6		2.4	
95% CI	4.9–7.7		1.9–2.6		1.1–3.4		2.1–4.0	
**OS, months**								
Median	14.5		6.3		4.6		8.1	
95% CI	12.6–16.2		4.6–8.4		1.3–21.2		5.3–16.9	
**RR, %**								
	31.9		2.5		0		0	
95% CI	22.7–42.3		0.1–13.2		0.0–33.6		0.0–41.0	
**Response, No, %**								
CR	0	0	0	0	0	0	0	0
PR	30	31.9	1	2.5	0	0	0	0
Long SD >6 months	45	47.9	3	7.5	1	11.1	1	14.3

Abbreviations: CR=complete response; DCR=disease control rate; mut=mutant; NE=not evaluable; PD=progressive disease; PR=partial response; SD=stable disease; wild=wild-type.

Wild-type *RAS/BRAF* was defined as all wild-type sequences with *RAS, BRAF*^V600E^, and *BRAF*^non-V600E^.

**Table 4:** Univariate and multivariate analysis with RAS/BRAF as a covariate for OS and PFS

	**PFS**				**OS**			
	**Univariate**		**Multivariate**		**Univariate**		**Multivariate**	
	**HR**	***p***	**HR**	***p***	**HR**	***p***	**HR**	***p***
***RAS/BRAF***								
Mutant/wild-type	3.49	<0.0001	5.43	<0.0001	2.14	<0.0001	3.37	<0.0001
**Age**								
	0.98	0.0078	0.98	0.0547	0.98	0.0493	excluded	
**Gender**								
Female/male	1.27	0.1585	0.78	0.1852	1.48	0.0268	0.84	0.405
**ECOG PS**								
1,2/ 0	1.12	0.4919	1.17	0.3854	1.16	0.3979	1.06	0.7609
**Histology**								
Well/others	1.93	0.1471	3.12	0.021	1.99	0.159	2.58	0.0597
Moderate/others	1.79	0.169	4	0.0028	1.86	0.179	3.28	0.0139
Poor/others	1.6	0.3193	2.6	0.0691	2.32	0.1	4	0.009
**Primary site**								
Right-sided/others	1.3	0.1974	1.52	0.0641	1.38	0.1335	1.73	0.0213
**Resection**^a^								
Yes/no	0.91	0.6776	excluded		0.74	0.2038	excluded	
**Adjuvant chemotherapy**								
Yes/no	0.87	0.4153	0.7	0.048	0.5	0.0036	0.64	0.1202
**Metastasis**								
Synchronous/metachronous	0.87	0.4153	0.7	0.048	0.62	0.0058	0.43	0.0001
**Combined with irinotecan**								
Yes/no	0.69	0.0649	0.52	0.0042	0.67	0.0524	0.42	0.0004
**Prior Ox**^b^								
Intolerance/failure	0.55	0.0322	0.63	0.1322	0.53	0.0383	0.38	0.0035

Abbreviations: ECOG=Eastern Cooperative Oncology Group; moderate=moderately differentiated; poor=poorly differentiated; PS=performance status; well=well differentiated.

a Primary tumour resection.

b Prior oxaliplatin treatment; wild-type *RAS/BRAF* was defined as all wild-type sequences with *RAS*, *BRAF*^V600E^, and *BRAF*^non-V600E^.
