# Supplementary material for: Clinical significance of BRAF non-V600E mutations on the therapeutic effects of anti-EGFR monoclonal antibody treatment in patients with pretreated metastatic colorectal cancer: the Biomarker Research for anti-EGFR monoclonal Antibodies by Comprehensive Cancer genomics (BREAC) study
Source: Br J Cancer. 2017 Oct 3;117(10):1450–8. doi: 10.1038/bjc.2017.308 (PMC5680457; doi:10.1038/bjc.2017.308)
Supplement: Supplementary Appendix [file bjc2017308x1.docx]

***Supplementary Appendix***

**Materials and Methods**

*The comprehensive genomics in the exploratory cohort*

The candidate biomarkers were explored in the exploratory cohort using the genetic technologies of whole exon sequencing, genome-wide association studies (GWASs), single nucleotide polymorphism (SNP) arrays, and copy number variation (CNV) analyses for the strictly selected cases with formalin-fixed, paraffin-embedded (FFPE) archival clinical samples in both cancerous and noncancerous areas, and blood samples if available.

*Selection criteria for the inference cohort*

The main inclusion criteria in the inference cohort were as follows: histologically confirmed adenocarcinoma of colon or rectum; unresectable metastatic disease; wild-type or unknown *KRAS* exon 2 status; at least 20 years of age; Eastern Cooperative Oncology Group (ECOG) performance status (PS) of 2 or less; at least one measurable lesion; documented refractory or intolerant to prior fluoropyrimidines, oxaliplatin, and refractory to prior irinotecan; received either cetuximab or panitumumab monotherapy or in combination with irinotecan since June 2010; baseline computed tomography (CT) performed within 42 days before initial administration of anti-EGFR antibody treatment; initial CT evaluation performed within 3 months after initial administration of anti-EGFR antibody treatment; adequate archival formalin-fixed and paraffin-embedded (FFPE) tissue specimens available; and adequate organ function (white blood cell count ≥ 2,000/mm^3^ and < 12,000/mm^3^, platelets ≥ 75,000/mm^3^, haemoglobin ≥ 8.0 g/dL, total bilirubin ≤ 3 × upper limit of normal [ULN], aspartate aminotransferase [AST] and alanine aminotransferase [ALT] ≤ 3 × ULN, and creatinine ≤ 2 × ULN. Main exclusion criteria included the presence of concurrent other active cancer, co-existing severe infection, fluid collection requiring drainage, and prior and/or co-existing interstitial pneumonitis.

*Preparation of clinical samples and DNA extraction*

Archived FFPE tissue specimens collected before administration of anti-EGFR antibody treatment in both cancerous areas and noncancerous areas were used for the analysis. FFPE tissue sections cut from either biopsied or surgically resected specimens were submitted to the Division of Translational Research, EPOC. The histology of tissue samples submitted from multi-institutions was confirmed by the certificated pathologist (S.F.) for central pathology diagnosis. Cancer-derived DNA and normal DNA were then extracted from microscopically or macroscopically dissected regions of the cancer area or the normal tissue area respectively by using an Absolutely RNA FFPE kit (modified protocol for DNA extraction; Agilent Technologies, Santa Clara, CA, USA). DNA quality was determined using a NanoDrop 2000 spectrophotometer (Thermo Fisher Scientific, Waltham, MA, USA), Quant-iT PicoGreen dsDNA Reagent and Kit (Life Technologies, Carlsbad, CA, USA), and Infinium HD FFPE DNA Sample QC Kit (Illumina, San Diego, CA, USA).

*Identification of SNVs and INDELs*

Sequence reads were aligned to the human reference genome UCSC hg19 using the Burrows-Wheeler Aligner program (BWA, http://bio-bwa.sourceforge.net/)^1^, and SNVs and INDELs were called and annotated using the Genome Analysis Toolkit software package (GATK, http://www.broadinstitute.org/gatk/)^2^. Sequencing artefacts were filtered out using custom filters (GATK confidence score ≥ 50, number of variant reads in each direction ≥ 1, variant allele frequency ≥ 10%). Germline variants were filtered out using data from dbSNP build 131, the 1000 Genomes Project (Phase 1 exome data, released 20110521), one Japanese genome, and 299 in-house Japanese exomes.

*Targeted resequencing*

Using 0.1–1.0 μg double-stranded DNA, we prepared the target-sequencing libraries. The probes were designed for a final capture size of 192.2 kb using the SureDesign system (Agilent Technologies, Santa Clara, CA, USA). Targeted sequence enrichment was performed using the Agilent SureSelect Target Enrichment Kit (Agilent Technologies), according to the manufacturer’s instructions. The capture libraries were sequenced using HiSeq 2000 or 1500 systems (Illumina, San Diego, CA, USA) to generate 100-bp paired-end data.

*BRAF activity assay*

To clarify the activity of novel BRAF mutants, HEK293 cells, which stably expressed wild-type *EGFR* cDNA (Mimaki, unpublished data), were transiently transfected with FLAG-BRAF-wild-type, -V600E, -Q524L, or -L525R expression vectors using FuGene HD transfection reagent (Promega, Madison, WI, USA). Detailed information about the plasmid vectors can be provided upon request. Twenty-four hours after transfection, cells transfected with BRAF-wild-type, -V600E, -Q524L, or -L525R were treated with the indicated concentration of cetuximab (MerckSerono, Geneva, Switzerland) for 2 h. Total cell lysates were applied for Western blotting using anti-FLAG, anti-ERK, and anti-phospho-ERK antibodies. Monoclonal anti-FLAG M2 antibodies were purchased from Sigma-Aldrich (St. Louis, MO, USA). Other antibodies were purchased from Cell Signaling Technologies (Danvers, MA, USA). The images and intensities of the blots were evaluated using an ImageQuant LAS 4000 mini lumino-image analyzer (GE Healthcare, Little Chalfont, UK). The phosphorylation status of ERK was evaluated by determining the intensity of phospho-ERK relative to that of ERK and standardized according to the intensity of exogenously expressed FLAG-BRAF.

Supplementary References

1. Li H, Durbin R: Fast and accurate short read alignment with Burrows-Wheeler transform. Bioinformatics 25:1754-1760, 209.
2. McKenna A, Hanna M, Banks E, et al: The Genome Analysis Toolkit: a MapReduce framework for analyzing next-generation DNA sequencing data. Genome Res 20:1297-1303, 2010.

**Supplementary Figure 1.**

Representative cases of super-responders and nonresponders in the exploratory cohort

Super-responders were defined as patients who showed a partial or complete response and/or progression-free survival for more than 6 months, while nonresponders were defined as those who showed a progressive disease at the first imaging within 3 months, when the relative dose intensity of anti-EGFR antibody treatment is more than 80%. Oncologists and radiologists from all participating institutions reviewed the results of the baseline and follow-up computed tomography (CT) of all the patients to evaluate the antitumour activity of anti-EGFR antibody treatment. After discussion, the professionals classified the patients in the exploratory cohort as super-responders or nonresponders.

**Supplementary Figure 2. Diagram in exploratory cohort**

**Supplementary Figure 3. Diagram in inference cohort**

**Supplementary Figure 4.**

Exploratory overall survival (OS) curves after anti-EGFR antibody treatment divided by primary tumour location in patients with pretreated mCRC according to mutational status. OS curve transitions from patients with wild-type *RAS* (n = 110) to patients with the *RAS*/*BRAF*^V600E^ wild-type genotype (n = 101) and to patients with wild-type *RAS*/*BRAF* (n = 94) were observed. The clear differences in OS between the right-sided colon and left-sided colon or rectum in patients with wild-type *RAF* became smaller when limited to wild-type *RAS/BRAF*; that is, there were no clear differences in OS among sites of primary lesions.
