# Supplementary material for: Clinical significance of BRAF non-V600E mutations on the therapeutic effects of anti-EGFR monoclonal antibody treatment in patients with pretreated metastatic colorectal cancer: the Biomarker Research for anti-EGFR monoclonal Antibodies by Comprehensive Cancer genomics (BREAC) study
Source: Br J Cancer. 2017 Oct 3;117(10):1450–8. doi: 10.1038/bjc.2017.308 (PMC5680457; doi:10.1038/bjc.2017.308)
Supplement: Supplementary Information [file bjc2017308x2.docx]

**
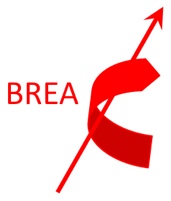
**

**Biomarker Research for**

**Anti-EGFR Monoclonal Antibodies**

**using Comprehensive Cancer Genomics**

**Protocol**

**[Principal Investigator]**

Atsushi Ohtsu

Director of Exploratory Oncology Research and Clinical Trial Center, National Cancer Center

6-5-1 Kashiwanoha, Kashiwa, Chiba 277-8577 Japan

Yukiko Abe

President of G&G SCIENCE CO., LTD.

4-1-1 Misato, Matsukawa-machi, Fukushima-shi, Fukushima

**[Contact Information]**

Katsuya Tsuchihara（Genetic data management department）

Division of Translational Research, Exploratory Oncology Research and Clinical Trial Center, National Cancer Center

6-5-1 Kashiwanoha, Kashiwa, Chiba 277-8577 Japan

Takayuki Yoshino（Specimen and clinical data management department）

Department of Gastroenterology and Gastrointestinal Oncology, National Cancer Center Hospital East

6-5-1 Kashiwanoha, Kashiwa, Chiba 277-8577 Japan

Protocol version 1.0　 October 25, 2011

Protocol version 1.1 November 23, 2011

Protocol version 1.2 December 14, 2011

Protocol version 1.3 October 26, 2012

Protocol version 1.4 August 5, 2014

**0.　Summary**

**0.1　Purpose**

To explore new biomarkers that predict clinical efficacy of an anti-epidermal growth factor receptor (anti-EGFR) antibody in patients with unresectable, metastatic colorectal cancer (CRC).

The investigation is conducted by whole exon analysis, genome-wide association study (GWAS), and copy number variation (CNV) analyses with single nucleotide polymorphism (SNP) arrays. Genomic DNA extracted from FFPE (formalin fixed paraffin embedded) samples from both a cancerous tissue and a non-cancerous one, as well as blood samples from enrolled subjects who received anti-EGFR antibodies are used. The purpose is to identify somatic mutations and SNPs related to therapeutic efficacy or serious adverse events, and to evaluate correlations between their existence and therapeutic efficacy in the enrolled subjects using a statistical method.

**0.2　Patient enrollment**

**0.2.1　Eligibility criteria**

**[Exploratory cohort]**

1) Histologically confirmed primary adenocarcinoma of the colon or rectum.

2) Unresectable advanced recurrent CRC.

3) KRAS genotype is wild-type or unknown (information may become available after commencing therapy).

4) CT scan at baseline has been conducted within 42 days before commencing therapy.

5) Has at least one measurable lesion.

6) Received therapy with medications including cetuximab, between September 2008 and May 2010.

7) Has at least one diagnostic imaging performed within 3 months after commencing therapy with medications including cetuximab.

8) Fluoropyrimidine resistant or has difficulty in re-administration.

9) Irinotecan resistant.

10) Oxaliplatin resistant or has difficulty in re-administration.

11) Aged 20 and above.

12) Sufficient FFPE samples.

13) Patients who received either cetuximab monotherapy or irinotecan-based chemotherapy combined with cetuximab.

14) Patients who fall in one of the following categories:

Super-responder: Cases with PR or more, or with 6 months or more of PFS according to RECIST guidelines (Version 1.1);

Non-responder: Cases in which the initial evaluation was PD, although drug intensity was retained at 80% or more according to RECIST guidelines (Version 1.1);

Cases that developed adverse events: allergy of grade 3, interstitial pneumonitis, skin rash of grade 3 within 2 weeks, or cases that developed unexpected serious adverse events.

**[Inference cohort]**

1) Histologically confirmed primary adenocarcinoma of the colon or rectum.

2) Unresectable advanced recurrent CRC.

3) *KRAS* genotype is wild-type or unknown (information may become available after commencing therapy)

4) CT scan at baseline has been conducted within 42 days before commencing therapy.

5) Has at least one measurable lesion.

6) Received therapy with medications including cetuximab or panitumumab between June 2010 and the date of approval by the institutional review board from each institution.

7) Has at least one diagnostic imaging performed within 3 months after commencing therapy with medications including cetuximab or panitumumab.

8) Fluoropyrimidine resistant or has difficulty in re-administration.

9) Irinotecan resistant.

10) Oxaliplatin resistant or has difficulty in re-administration.

11) Aged 20 and above.

12) PS: 0 to 2 (ECOG performance status score).

13) Function of major organs is observed as follows by a test just before administration of cetuximab or panitumumab :

(1) White blood cell count: ≥ 2000/mm^3^, < 12 000/mm^3^;

(2) Platelet count: ≥ 75 000/mm^3^;

(3) Hemoglobin: ≥ 8.0 g/dL;

(4) Total serum bilirubin: ≤ 3 × upper limit of normal (ULN);

(5) AST (GOT) and ALT (GPT): ≤ 3 × ULN
(Patients with liver metastasis should be ≤ 5 × ULN);

(6) Total serum creatinine: ≤ 2 × ULN.

14) Sufficient FFPE samples.

15) Received cetuximab monotherapy, irinotecan-based chemotherapy combined with cetuximab, panitumumab monotherapy, or irinotecan-based chemotherapy combined with panitumumab.

**0.2.2　Exclusion criteria**

**[Exploratory cohort]**

Exclude patients who satisfy the following exclusion criteria at a test before commencing therapy with medications including cetuximab:

(1) A patient who has active double cancer.

(2) A patient whom an investigator has determined to be unfit for the study.

(3) A patient who refuses in writing to allow his/her samples to be used for research.

**[Inference cohort]**

Exclude patients who satisfy the following exclusion criteria at a test before commencing therapy with medications including cetuximab or panitumumab:

(1) A patient who has active double cancer.

(2) A patient who has a complication from a serious infectious disease.

(3) A patient who requires treatment due to body cavity fluid accumulation (Pleural effusion, ascites, or pericardium effusion).

(4) A patient with a history or current diagnosis of interstitial pneumonitis and/or pulmonary fibrosis.

(5) A patient whom an investigator has determined to be unfit for the study.

(6) A patient who refuses in writing to allow his/her samples to be used for research.

**0.3　Methods of the study**

**0.3.1　Samples and their retrieval**

Investigators in each institution^*^, *3 prepare FFPE samples from cancerous and non-cancerous tissues, which are thin-sectioned to 10 μm (n = 10) and 2 μm (n = 5) samples (See the attached document, “Standard operation procedure of pathology”). Prepared FFPE samples are given an institutional case number, the personal information of patients is encrypted, and the FFPE samples with institutional case numbers are collected by SRL, Inc. (SRL). SRL prepares hematoxylin and eosin (HE) stained slides of cancerous or non-cancerous tissue from the FFPE samples. In addition, a collaborator in each institution*3 collects 5 mL of blood in an EDTA 2Na-containing blood collection tube from a patient who is able to provide a blood sample. The blood samples are given the same institutional case numbers as the FFPE samples, the personal information of the patients is encrypted, and the numbered blood samples are collected by SRL. SRL extracts DNA from the blood samples, with each genomic DNA identified by an institutional case number. SRL sends HE-stained slides and remaining unstained slides, as well as extracted DNA to the Center for Research Administration and Support of the National Cancer Center. From there, FFPE samples and genomic DNA with the institutional case numbers are delivered to a personal information manager at the National Cancer Center Hospital East, who replaces the institutional case numbers with new sample codes. Finally, after linkable anonymization, the FFPE samples and genomic DNA with sample codes are delivered to the research office (See the attached document, “Operation procedure of collection of samples.”).

*These are the institutions involved.

Department of Gastroenterology and Gastrointestinal Oncology, National Cancer Center Hospital East

Department of Gastroenterology and Hepatology, Hokkaido University Hospital

Division of Gastrointestinal Oncology, Shizuoka Cancer Center

Department of Gastroenterology, Cancer Institute Hospital

Department of Gastroenterology, Saitama Cancer Center

Department of Clinical Oncology, Aichi Cancer Center Hospital

Department of Internal Medicine, Shikoku Cancer Center

　*1: Handling of samples by the Shizuoka Cancer Center is organized as follows.

　G&G SCIENCE CO., LTD., which is a principal corporate research participant in the study, establishes a laboratory, dispatches researchers within the Shizuoka Cancer Center Research Institute, and prepares a collaboration contract that covers also security issues. G&G SCIENCE CO., LTD. performs research, such as genetic analysis, in accordance with paragraph “7.4.5 Protection of privacy and handling of patient information.”

SRL sends HE-stained slides and remaining unstained slides, as well as extracted DNA, to a personal information manager at the Shizuoka Cancer Center, who deletes the institutional case number and gives a new sample code, and after linkable anonymization, delivers the FFPE samples and genomic DNA with sample codes to the principal investigator at the Shizuoka Cancer Center (see the attached document, “Operation procedure of collection of samples.”).

**0.3.2　Collection of clinical data^*6^**

Investigators in each institution ^*2^ confirm the eligibility of study patients and fill in the case report form (CRF) using the web data capture system. Only clinical data should be entered in the attached documents: web-CRF(1) Eligibility confirmation form, web-CRF(2) Research on details of patient background and information on efficacy and safety, and web-CRF(3) Additional research on efficacy and safety. Until completion of the web enrollment system, paper CRFs are filled in and faxed to the Clinical Trial Support Office, National Cancer Hospital East. To avoid the risk of leaking personal information of the patients, an original institutional case number should be used in place of the chart numbers of each institution.

The Center for Research Administration and Support of the National Cancer Center verifies that no clinical data associated with the institutional case number has been deleted. If there is a deletion, the form is returned to a collaborator in each institution before any linkable anonymization is ordered from a personal data manager. If there is no deletion, the clinical data associated with the institutional case number is delivered to the personal data manager. The personal data manager deletes the institutional case number and assigns the same sample code as for the corresponding sample, and after linkable anonymization, delivers the clinical data associated with the sample code to the research office. Database preparation and data management are carried out at the Center for Research Administration and Support of the National Cancer Center.

Additional research on efficacy and safety is performed regularly (until March 31, 2016) only for patients who are undecided on any of the following: safety, response rate, progression-free survival, and overall survival, using web-CRF(3) Additional research on efficacy and safety. Linkable anonymization is similarly conducted as described in the above procedure.

The web enrollment URL is separately notified by the “Web enrollment system using manual.” (Enrollment is available for 24 hours; when not available due to maintenance or other causes, users are informed beforehand.)

*2: Handling of clinical data by Shizuoka Cancer Center is determined as follows.

A researcher at the Shizuoka Cancer Center confirms the eligibility of study patients and describes clinical data associated with the institutional case number in the attached documents: CRF(1) Eligibility confirmation form, CRF(2) Research on details of patient background and data on efficacy and safety, and CRF(3) Additional research on efficacy and safety. The researcher delivers them to the personal data manager at the Shizuoka Cancer Center, who replaces the institutional case number with the same sample code as the corresponding sample. After linkable anonymization, the clinical data associated with a sample code is delivered to the principal investigator at the Shizuoka Cancer Center, who sends it to the research office at the National Cancer Center by FAX. Database preparation and data management are conducted at the Center for Research Administration and Support of the National Cancer Center. To avoid the risk of leaking the personal data of patients, use of a chart number should be avoided.

Additional research on efficacy and safety is performed regularly (until March 31, 2016) only for patients who are undecided on any one of the following: safety, response rate, progression-free survival, and overall survival, using CRF(3) Additional research on efficacy and safety. Linkable anonymization is similarly conducted as described in the above procedure.

Contact address for web enrollment

Center for Research Administration and Support of the National Cancer Center

From 10 a.m. to 5 p.m. on weekdays (Closed on Saturdays, Sundays, and public holidays)

**0.3.3　Whole exon sequence analysis**

DNA is extracted from cancerous and non-cancerous tissues collected from all FFPE samples, as well as blood from all samples. The whole exon region is enriched using the target capture method. The aim is to sequence 10 depths or more, covering ≥ 90% of the whole exon, using a high-speed sequencer, such as the Illumina Genome Analyzer. A fluorescent beads array system is used to identify exons with a genomic DNA mutation or structural anomaly.

**0.3.4　GWAS analysis/CNV analysis**

GWAS and CNV analyses using a SNP array are performed using the Illumina iScan System. DNA is extracted from cancerous and non-cancerous tissues of all FFPE samples, as well as blood from all samples. DNA fragmented by formalin fixation is assessed by real time PCR and repaired prior to SNP genotyping by using the Infinium HD Assay. For FFPE samples, the aim is to obtain data for ≥ 90% of the call rate using Illumina’s HumanOmniExpress_FFPE-12, which is suitable for DNA extracted from formalin-fixed specimens. For blood samples, the aim is to obtain data for ≥ 99% of the call rate using HumanOmniExpress-12.

**0.3.5　Therapy evaluation and safety evaluation**

Investigators in each institution perform therapy evaluation in accordance with the RECIST guidelines (Version 1.1). A safety evaluation is conducted using CTCAE (Common Terminology Criteria for Adverse Events) Version 4.0, JCOG version translated in Japanese (CTCAE v4.0 - JCOG).

**0.3.6　Correlating DNA analysis with clinical data**

The correlation between genomic data and clinical response, or an adverse event following treatment with cetuximab or panitumumab, are analyzed using the biomarker exploratory cohort. The extracted gene groups are used in the inference cohort.

**0.4　Target sample size and research period**

**0.4.1　Target sample size**

　・Exploratory cohort sample：Maximum 150 cases（Patients who received therapy with medications including cetuximab, between September 2008 and May 2010）;

　・Inference cohort sample： 250 or more cases（Patients who received therapy with medications including cetuximab or panitumumab, between June 2010 and the date of approval by the institutional review board）.

**0.4.2　Research period**

From the date of approval by the institutional review board until March 31, 2016.

**0.5　Ethical issues**

**0.5.1　Ethical guidelines**

The study complies with the Declaration of Helsinki and the Ethical Guidelines for Human Genome/Gene Analysis Research, which is a joint guideline by three government ministries. Protection of the patients’ human rights and all matters concerning patients' agreement are set and approved by the institutional review board (IRB).

**0.5.2　Approval by the institutional review board**

The study is conducted after the protocol and the informed consent form are evaluated and approved by the IRB of each institution.

**0.6　Research funds and conflicts of interest**

The study is supported by research and development costs of Program 23-A-2 “Bridging basic and clinical research, promotion of translational research to implement personalized medicine” from the National Cancer Center in the fiscal year 2011; Japan Science and Technology Agency’s A-STEP (Adaptable and Seamless Technology Transfer Program through Target-driven R&D), practical application challenge type “Development of small- and medium-sized enterprises/venture businesses”; and research funds provided by G&G SCIENCE CO., LTD. and MEDICAL & BIOLOGICAL LABORATORIES CO., LTD. Japan Science and Technology Agency’s A-STEP (Adaptable and Seamless Technology Transfer Program through Target-driven R&D), practical application challenge type “Development of small- and medium-sized enterprises/venture businesses” is commissioned to G&G SCIENCE CO., LTD. from Japan Science and Technology Agency, therefore, management of research funding and research progress reports are operated by G&G SCIENCE CO., LTD. A part of the research effort is recommissioned from G&G SCIENCE CO., LTD. to each institution and funds are provided. MEDICAL & BIOLOGICAL LABORATORIES CO., LTD. is in charge of the administrative aspect of running a workshop, preparation of material for research, and contact points. No conflict of interest is declared in relation to this study. Any intellectual property that may arise from the study is managed under a separate collaborative research agreement.

**0.7　Contact Information**

Research office （Genetic data management department）Katsuya Tsuchihara

Division of Translational Research, Exploratory Oncology Research and Clinical Trial Center, National Cancer Center

6-5-1 Kashiwanoha, Kashiwa, Chiba 277-8577 Japan

Research office（Specimen and clinical data management department）

Takayuki Yoshino

Department of Gastroenterology and Gastrointestinal Oncology, National Cancer Center Hospital East

6-5-1 Kashiwanoha, Kashiwa, Chiba 277-8577 Japan

**Table of contents**

0. Summary 1

0.1　Purpose 1

0.2　Patients enrollment 1

0.3　Methods of the study 3

0.4　Target sample size and research period 7

0.5　Ethical issues 7

0.6　Research funds and conflicts of interest 8

0.7　Contact Information 8

1. Purpose　　　　　　　　　　　　　　　　　　　　 11

2. Background 11

2.1　Current status of personalized medicine 11

2.2　*KRAS* gene mutation and anti-EGFR antibodies 12

2.3　Current availability of biomarkers for anti-EGFR therapy 12

2.4　Identification of biomarkers using a comprehensive genetic analysis method 13

2.5　Reliability of clinical samples 14

2.6　Significance of the study 16

3．Criteria for selecting patients 17

3.1　Eligibility criteria 17

3.2　Exclusion criteria 　 19

4. Methods of the study 20

4.1　Preparation of FFPE samples at each institution 20

4.2　Analysis of the exploratory cohort 21

4.3　Analysis of the inference cohort 27

4.4　Correlating DNA analysis with clinical data 28

4.5　Flow of samples and genetic data 28

4.6　Flow of anonymization 31

5. Target sample size and research period 32

5.1　Target sample size 33

5.2　Research period 　　　　　　　　　　　　　　　 33

6．Statistical analysis 33

6.1　Analysis of the exploratory cohort 33

6.2　Analysis of the inference cohort 35

7．Ethical issues 37

7.1　Protection of patients 37

7.2　Ethical guidelines 38

　7.3　Approval by the institutional review board 38

7.4　Basic attitude toward patients 38

7.5　Methods for storing and discarding samples　 42

8. Presentation of results 43

9. Secondary use of data 4

10. Compliance with the protocol 44

11. Approval by the institutional review board 44

11.1　Approval at the beginning of study participation 44

11.2　Annual update of approval at each institution 45

11.3　Modification of the CRF 45

12. Record keeping 45

13. Report on discontinuation/completion of the clinical study 45

14. Research funds and conflicts of interest 45

15. Research organizations and researchers 45

16. References 45

**1. Purpose**

To explore new biomarkers that predict the clinical efficacy of an anti-epidermal growth factor receptor (anti-EGFR) antibody in patients with unresectable, metastatic colorectal cancer (CRC).

The investigation is conducted by whole exon analysis, genome-wide association study (GWAS), and copy number variation (CNV) analyses with single nucleotide polymorphism (SNP) arrays. Genomic DNA extracted from FFPE (formalin fixed paraffin embedded) samples from both cancerous and non-cancerous tissues, as well as blood samples from enrolled subjects who received anti-EGFR antibodies are used. The purpose is to identify somatic mutations and SNPs related to therapeutic efficacy or serious adverse events, and to evaluate therapeutic efficacy in the enrolled subjects using a statistical method.

**2. Background**

**2.1 Current status of personalized medicine**

The importance of personalized medicine, which considers a patient's individual physiological and pathological character, has been recognized. In molecular-targeted cancer therapy, maximum clinical efficacy and minimum adverse drug reactions are expected by tailoring the therapy to the patient. This can be achieved by using various biomarkers, including gene mutations, before administrating the therapeutic agent. Furthermore, personalized medicine offers not only advantages for patients but also significant cost savings. These include lower costs of therapeutic agents for which an effect is not expected, reduced costs for measures against adverse drug reactions, and improvements in public health.

In order to implement personalized medicine, the identification of biomarkers to predict clinical efficacy and confirm safety is necessary. The U.S. Food and Drug Administration (FDA) announced a draft guidance on the clinical application of biomarkers in July 2011.^1)^ Simultaneous proceedings for the development of both diagnostic and therapeutic agents using biomarkers are considered to be more generalized in the future.

In Japan, a notification on “ICH E16: Biomarkers related to drug or biotechnology product development: Context, structure and format of qualification submissions” was issued by the Director of the Evaluation and Licensing Division, and by the Director of the Safety Division, both of Pharmaceutical and Food Safety Bureau of the Ministry of Health, Labour and Welfare (MHLW) on January 20, 2011.^2)^ It allowed the use and validation of biomarkers (appropriateness of using the biomarker is supported by proposed data) at any time during research, development, or after approval of therapeutic agents. This encouraged pharmaceutical companies to acquire data on genomic biomarkers.

The development of biomarkers is necessary to provide maximum clinical efficacy and minimum adverse drug reactions, for newly developed drugs and previously commercialized ones. In addition, it helps bring down the costs.

**2.2 *KRAS* gene mutation and anti-EGFR antibodies**

At the annual meeting of the American Society of Clinical Oncology (ASCO) in 2008, the results of a retrospective analysis of Kirsten rat sarcoma (*KRAS*) genes in subjects of intention-to-treat (ITT) analysis and subjects with the wild-type or mutated form of *KRAS* were presented. Clinical trials (CRYSTAL study^3)^ and OPUS study^4)^) showed that the efficacy of cetuximab, an anti-EGFR antibody, was lost if *KRAS* gene was mutated.

These results highlighted the ineffectiveness of an anti-EGFR antibody treatment in patients with *KRAS* mutation. In response to this, existence of *KRAS* gene mutation has to be confirmed in prior to administration of anti-EGFR antibody in Europe.^5)^ In the U.S., the guidelines of the National Comprehensive Cancer Network (NCCN) recommended administration only to patients with wild-type *KRAS*, and the FDA added a description of *KRAS* gene mutation to the package insert of anti-EGFR antibodies in July 2009.

In Japan, the package insert of cetuximab was revised in March 2010, stating that eligible patients should be selected after considering the presence or absence of *KRAS* gene mutation. Similarly, panitumumab was approved for advanced/recurrent colorectal cancer with the wild-type *KRAS* gene in April 2010, and *KRAS* gene mutation began to be clinically used for patient selection for anti-EGFR antibodies.

**2.3 Current availability of biomarkers for anti-EGFR therapy**

Although detecting *KRAS* gene mutation is effective for excluding a group in which anti-EGFR antibody treatment is ineffective, the response rate of anti-EGFR antibody monotherapy in patients with the wild-type *KRAS* gene remains 20% to 30%. The research for biomarkers other than *KRAS* gene mutation focused on molecules downstream of EGFR, in the RAS/RAF/MAPK and PI3K/Akt signaling pathways. Consequently, patients with gene mutations of BRAF, *NRAS*, or *PI3K* were reported by several researchers as belonging to a treatment-ineffective group. Thus, the possibility to refine a treatment-effective group by detecting combinations of several biomarkers was suggested (Fig. 1).^6)^


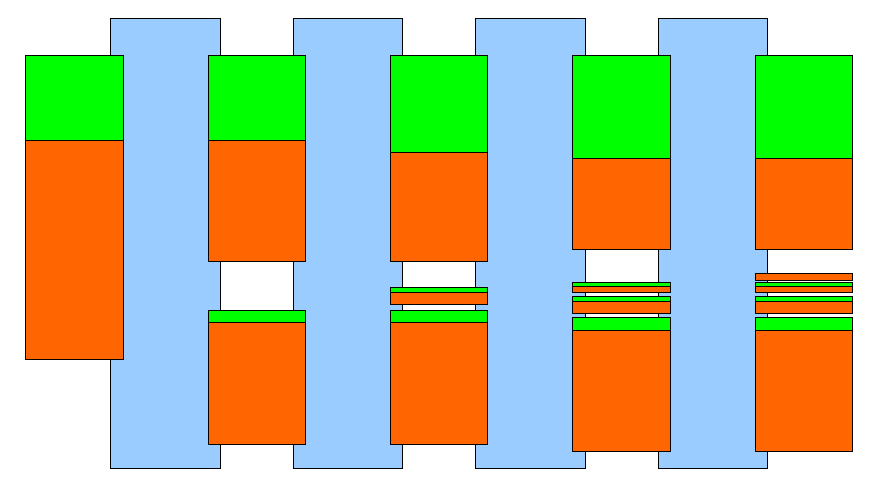


Study of *BRAF*

Response rate
36.3%

Study of *KRAS*

Response rate
24.4%

Mutation rate of
wild-type *KRAS/BRAF/NRAS* 3.1%

Mutation rate of wild-type *KRAS* 7.2%

Mutation rate 41.2%

Mutation rate of
wild-type *KRAS/BRAF* 4.8%

Response rate
38.4%

Study of *PI3K*

Study of *NRAS*

Response rate 41.2%

Response rate
39.9%

Figure 1. The improvement in response prediction gained by assessing the mutation status of each gene.

In addition, Sawyers et al. indicated that activity of NF-κB, which did not directly relate to the EGFR downstream signaling pathway, specifically affected the antitumor effect of small-molecule EGFR inhibitors.^7)^ These findings indicate that combining several new biomarkers has the potential of improving patient selection and the prediction of clinical efficacy of anti-EGFR antibodies against colon cancer in the future.

**2.4 Identification of biomarkers using a comprehensive genetic analysis method**

Genetic factors correlate deeply with individual differences in clinical efficacy or serious adverse events, and single nucleotide polymorphisms (SNP) are thought to be involved.^8)^ Generally, the role of SNPs in drug response is said to be relatively limited. However, accumulating evidence of remarkably treatment-effective groups and obviously non-effective groups, augments the possibility of identifying a SNP that predicts the therapeutic effect, even if the number of subjects is limited.^9, 10)^ A collaborator of the study, Mizokami et al., from the National Center for Global Health and Medicine, found an IL-28B-related SNP as a biomarker for predicting the effect of interferon therapy on hepatitis C using GWAS analysis. Thus, an SNP array with 900,000 SNPs, was tested in a relatively restricted number of patients, 64 of whom showed complete response to previously administered interferon/ribavirin therapy, and 78 who showed no response to the same therapy.^11)^

Since somatic mutations play an important role in the development and progression of cancer, they should be a major source of biomarkers for predicting therapeutic efficacy. In order to identify a variety of somatic mutations in cancer cells, whole-genome sequencing using the latest high-speed sequencer was performed, and various projects, such as the International Cancer Genome Consortium (ICGC), are proceeding. Recently, the whole-genome sequencing of a case of hepatoma by a group from the National Cancer Center reported was heralded as an achievement in Japan.^12)^ In addition, a group from the Dana-Farber Cancer Institute identified 11 translocations that developed into fusion genes by searching the genetic alteration of colorectal tumors in 9 patients and comparing them with normal tissues using a whole genome sequence analysis technique.^13)^

On the other hand, whole exon sequencing that only decodes the exon region, which corresponds to about 1% to 2% of the whole genome, has emerged as a more rapid method to analyze mutations related to cellular function. In 2011, as a result of whole exon sequencing of dozens to hundreds of cancers, profiles of specific gene mutations were reported in serosanguineous ovarian adenocarcinoma^14)^, and head and neck squamous cell carcinoma.^15), 16)^ In our preceding study at the Clinical Development Center of the National Cancer Center Hospital East, we conducted whole exon sequencing on 97 cases of resected specimens of lung adenocarcinomas and efficiently identified gene mutations observed in the cancer tissue. In addition, in the course of the study we developed a method that could efficiently remove errors from the vast amount of sequence data obtained from whole exon sequencing, and filed for a patent called “A large-scale base sequence analysis method, a program, and a device” (Japanese Patent Application No. 2011-105868).

From results of past large-scale sequence analyses and our preceding study, it has been determined that there are dozens to hundreds of mutations in somatic cells, including exons, per case of solid cancer, and most of them are not duplicated between samples. Through analysis of whole exon sequencing, it is important to identify gene mutations related to the therapeutic effect, consider these characteristics, and annotate functions from which to extract biological data.

In our preceding study based on the above, we successfully extracted several known and novel cancer-related genes, as well as pathways.

**2.5 Reliability of clinical samples**

The cases to use for the biomarker exploratory cohort or the inference cohort should be coupled with clinical data that can appropriately determine whether a group has an effective or ineffective response to cetuximab or panitumumab therapy. This should be accompanied by clinical data of serious adverse events, and the purity and degrees of fragmentation of genomic DNA obtained from samples of selected cases should be appropriate for analysis. If the above is not secured, highly reliable analytical results cannot be obtained by conducting any comprehensive genetic analyses. The medical oncologists of the study group have participated in many multinational studies and have had experience in the management and storage of samples and the planning and execution of data evaluation of clinical studies. This study will be carried out fully utilizing their expertise. Since the method of selecting cases that were either effective or ineffective to cetuximab or panitumumab therapy was also evaluated in a similar manner in clinical cases in the preceding study by the study group, rapid accumulation and analysis of the cases is considered possible in this study.

**2.6 Significance of the study**

**2.6.1 Significance of conducting the study**

The morbidity of colorectal cancer has been increasing in Japan recently, and this cancer type has the second-highest morbidity in both men and women. The number of patients was 99,770 and the mortality rate was 78.2/100,000 people in 2003. The number of deaths in 2007 was 42,172 people and, according to a recent international comparison, Japanese mortality rate was similar to Western Caucasians for both colon cancer and rectal cancer.

In advanced/recurrent rectal cancer, the median survival time (MST) is reported as about 8 months without chemotherapy, or about 2 years if chemotherapy is applied. Treatment with FOLFOX (oxaliplatin + continuous intravenous infusion of 5-FU), CapeOX (oxaliplatin + capecitabine), and FOLFIRI (irinotecan + continuous intravenous infusion of 5-FU) have been used as standard chemotherapy against advanced/ recurrent colorectal cancer. Molecular target drugs such as bevacizumab (anti-vascular endothelial growth factor [anti-VEGF] antibody), cetuximab (anti-EGFR antibody), and panitumumab (anti-EGFR antibody) are used. Recently, research on biomarkers for anti-EGFR antibodies has progressed, and personalized therapy has become possible. It has been said that the therapeutic effects of anti-EGFR antibodies were poor if *KRAS* gene was mutated. Since insurance reimbursement for *KRAS* gene testing was started in Japan in April 2010, absence of *KRAS* gene mutation is now confirmed before using anti-EGFR antibodies.

In Japan, anti-EGFR antibodies are mainly used with irinotecan as a third therapy. This is attributable to the fact that there is no clinical outcome demonstrating that anti-EGFR antibodies are surpassed by anti-VEGF antibodies for efficacy. Moreover, even if a case is refractory for irinotecan, the outcome of anti-EGFR antibody monotherapy has been reported to surpass that of combination therapy with an anti-EGFR antibody and irinotecan. Thus, in the study, patients who received monotherapy with either cetuximab or panitumumab, as well as those who received irinotecan-based chemotherapy combined with cetuximab or panitumumab, were considered as a population, in which an accumulation of many cases was expected. This includes anti-EGFR antibody effective or ineffective groups, and cases that developed serious adverse events by anti-EGFR antibodies in the homogeneous population with the pretreatment as a characteristic case.

In a study in which the cost-effectiveness of *KRAS* and *BRAF* gene testing for patient selection of the anti-EGFR antibody was estimated, the benefits of gene testing were indicated for *KRAS* and *BRAF* wild-type patients.^17)^ In addition, if patients for whom the therapeutic effect with the anti-EGFR antibody is high are selected by a biomarker other than *KRAS*, the costs of the anti-EGFR treatment can be reduced and the economic benefits become important. If the number of subjects who receive anti-EGFR antibodies (assumed to be 40,000 people) is reduced from 60% (the proportion of patients with wild-type *KRAS*) to 40% by utilizing biomarkers that refine the treatment-effective group, the cost-reduction effect of the anti-EGFR antibody reaches 14.4 billion yen per year (600,000 yen/month × 3 months of administration × 8,000 people).

If a biomarker that predicts the therapeutic effect of an anti-EGFR antibody is identified by the study, patients who receive great benefits from such treatment can be selected more easily with reducing patient burden, costs, and improving public health.

**2.6.2 Samples required for executing the study**

In this study, a comprehensive genetic analysis will be conducted in patients who are enrolled in the exploratory cohort (patients who respond to cetuximab, no response to cetuximab, and those who develop serious adverse events against cetuximab), and the use of candidates with biomarkers will be considered for enrollment in the inference cohort. An effective case from each institution will comprise 50 super-responder cases, which include CR,^*1^ PR, or cases for which ≥ 6 months of PFS is obtained. For an ineffective case, a total of 50 cases, which are determined as PD by the initial efficacy evaluation, will be accumulated from each institution. Similarly, a total of 50 cases that develop serious adverse events, such as allergy of grade ≥ 3, interstitial pneumonitis, skin rash of grade ≥ 3 within 2 weeks, or unexpected serious adverse events will be summed from each institution.

In order to collect 50 cases that show super-responder, non-responder, or develop serious adverse events each, a database of an estimated 500-case scale cetuximab-administered patient group is required.

Even at the study organization, which has the most abundant therapeutic experience in Japan, a 500-case scale prospective clinical study requires a total of 6 years (around 5 years of enrollment period and around one year of observation period). For this study which is a retrospective study, by tightening the eligibility criteria and strictly narrowing down the cases which were treated by medical oncologists at leading Japanese institutions, high-quality clinical data have been accumulated and about 500 cases have already been secured. By using patients who have already shown the clinical efficacy of cetuximab, the case enrollment period can be drastically shortened, making earlier identification of a novel biomarker possible. Novel biomarker candidates are checked by cross validation of patients enrolled in the inference cohort (250 cases or more).

The study is useful for patients with colon cancer, not only in Japan, but also across the world; therefore, it is necessary to produce results as early as possible. On the other hand, the protection of human rights of patients in genomic/genetic research must be respected.

In the study, if consent can be obtained from patients, samples delineated in the consent form should be used. However, if consent from the patient or the patient's legally acceptable representative is difficult to obtain, we follow 7.4.2 “Response to a person from whom informed consent is difficult to obtain” and strictly comply with the Ethical Guidelines for Human Genome/Gene Analysis Research, and handle resected specimens of past colon cancer surgery and clinical data as a Group C human specimen. Samples such as Group C human specimens handled in the study are used for analysis after linkable anonymization, for which the researchers do not have the correspondence table because the specimens had been previously collected. This makes risks or disadvantages for patients and their family members minimal.

*1 CR: Complete Response

Complete disappearance of all target lesions and a minor axis of lymph nodes less than 10 mm, regardless of whether it is target or non-target.

**3. Criteria for selecting patients**

**3.1 Eligibility criteria**

**[Exploratory cohort]**

1) Histologically confirmed primary adenocarcinoma of the colon or rectum.

2) Unresectable advanced recurrent CRC.

3) *KRAS* genotype is wild-type or unknown (data may become available after commencing therapy).

4) CT scan at baseline has been conducted within 42 days before commencing therapy.

5) Has at least one measurable lesion.

6) Received therapy with medications including, cetuximab, between September 2008 and May 2010.

7) Has at least one diagnostic imaging performed within 3 months after commencing therapy with medications including cetuximab.

8) Fluoropyrimidine resistant or has difficulty in readministration.

9) Irinotecan resistant.

10) Oxaliplatin resistant or has difficulty in readministration.

11) Aged 20 and above.

12) Sufficient FFPE samples.

13) Patients who received either cetuximab monotherapy or irinotecan-based chemotherapy combined with cetuximab.

14) Patients who fall in one of the following categories:

Super-responder: Cases with PR or more, or with ≥ 6 months of PFS according to RECIST guidelines (Version 1.1);

Non-responder: Cases in which the initial evaluation was PD, although drug intensity was retained at ≥ 80% according to RECIST guidelines (Version 1.1);

Cases that developed adverse events: Allergy and interstitial pneumonitis of grade ≥ 3, skin rash of grade 3 within 2 weeks, or cases that developed unexpected serious adverse events.

**[Inference cohort]**

1) Histologically confirmed primary adenocarcinoma of the colon or rectum.

2) Unresectable, advanced recurrent CRC.

3) *KRAS* genotype is wild-type or unknown (data may become available after commencing therapy)

4) CT scan at baseline has been conducted within 42 days before the start of therapy.

5) Has at least one measurable lesion.

6) Received therapy with medications including cetuximab or panitumumab between June 2010 and the date of approval by the IRB from each institution.

7) Has at least one diagnostic imaging performed within 3 months after commencing therapy with medications including cetuximab or panitumumab.

8) Fluoropyrimidine resistant or has difficulty in readministration.

9) Irinotecan resistant.

10) Oxaliplatin resistant or has difficulty in readministration.

11) Aged 20 and above.

12) PS: 0 to 2（ECOG performance status score）.

13) Function of major organs is observed as follows by a test just before the administration of cetuximab or panitumumab:

(1) White blood cell count: ≥ 2000/mm^3^, < 12,000/mm^3^;

(2) Platelet count: ≥ 75,000/mm^3^;

(3) Hemoglobin: ≥ 8.0 g/dL;

(4) Total serum bilirubin: ≤ 3 × upper limit of normal (ULN);

(5) AST (GOT) and ALT (GPT): ≤ 3 × ULN
(Patients with liver metastasis should be ≤ 5 × ULN);

(6) Total serum creatinine: ≤ 2 × ULN.

14) Sufficient FFPE samples.

15) Received cetuximab monotherapy, irinotecan-based chemotherapy combined with cetuximab, panitumumab monotherapy, or irinotecan-based chemotherapy combined with panitumumab.

**3.2 Exclusion criteria**

**[Exploratory cohort]**

Exclude patients who satisfy the following exclusion criteria at a test before commencing therapy with medications including cetuximab:

(1) A patient who has active double cancer.

(2) A patient whom an investigator has determined to be unfit for the study.

(3) A patient who refuses in writing to allow his/her samples to be used for research.

**[Inference cohort]**

Exclude patients who satisfy the following exclusion criteria at a test before commencing therapy with medications including cetuximab or panitumumab:

(1) A patient who has active double cancer.

(2) A patient who has a complication from serious infectious disease.

(3) A patient who requires treatment due to body cavity fluid accumulation (Pleural effusion, ascites, or pericardium effusion).

(4) A patient with a history or current diagnosis of interstitial pneumonitis and/or pulmonary fibrosis.

(5) A patient whom an investigator has determined to be unfit for the study.

(6) A patient who refuses in writing to allow his/her samples to be used for research.

**4. Methods of the study**

**4.1 Preparation of FFPE samples at each institution**

**4.1.1 Preparation from surgical specimens**

(1) Method for selecting the most appropriate section of a cancerous tissue.

Sections that satisfy the following conditions as much as possible are recommended.

1. Cancerous cells that exist regionally with high density and where the section corresponds to the maximum diameter of the cancer.

2. Exclude factors that make it difficult to extract only cancer cells and amplify the DNA as much as possible.

Factors: Necrosis, mucin lake formation, invasion of inflammatory cells to cancerous cells, and enlarged tumor stroma.

3. Exclude a section that includes parts of morphologically obviously degenerated cells in a patient who had received chemotherapy or radiation therapy before surgery.

(2) Method of selecting the most appropriate section of a non-cancerous tissue.

Select a section that corresponds to either one of those below.

1. For surgical specimens of a primary lesion of the large intestine, usually a block of resection stump is prepared from either the actinal or the anal side, from which thin sections of non-cancerous tissue are derived.

2. For a metastatic lesion, non-cancerous tissue is usually present in a paraffin block that encompasses the maximum diameter of the tumor; therefore, the paraffin block is also used for non-cancerous tissue.

3. If there is no paraffin block corresponding to 1 and 2 above, prepare thin sections from a paraffin block from the lymph node without metastasis.

**4.1.2 Preparation from a biopsy tissue sample**

For a case where only biopsy tissue is collected, prepare as many thin sections as possible of both cancerous and non-cancerous tissues.

**4.2 Analysis of the exploratory cohort**

**4.2.1 Samples and their retrieval**

(1) Collection of FFPE samples.

Investigators at each institution^*3^ prepare FFPE samples from cancerous and non-cancerous tissues that are thin-sectioned to 10 μm (n = 10) and 2 μm (n = 5) samples (See the attached document, “Standard operation procedure of pathology”). FFPE samples are given an institutional case number, the personal data of patients is encrypted, and the FFPE samples with institutional case numbers are collected by SRL, Inc. (SRL). SRL prepares hematoxylin and eosin (HE) stained slides of cancerous or non-cancerous tissue from the FFPE samples. SRL sends the FFPE samples with the institutional case numbers (HE-stained slides and remaining unstained slides) to the Center for Research Administration and Support of National Cancer Center (See the attached document, “Operation procedure of collection of samples”). From there the FFPE samples with the institutional case numbers are delivered to a personal data manager at the National Cancer Center Hospital East, who replaces the institutional case numbers with new sample codes. Finally, after linkable anonymization, the FFPE samples with the sample codes are delivered to the research office (See the attached document “Flow chart of the study, No.1 [FFPE sample].”)

*:*These are the institutions involved.

Department of Gastroenterology and Gastrointestinal Oncology, National Cancer Center Hospital East

Department of Gastroenterology and Hepatology, Hokkaido University Hospital

Division of Gastrointestinal Oncology, Shizuoka Cancer Center

Department of Gastroenterology, The Cancer Institute Hospital

Department of Gastroenterology, Saitama Cancer Center

Department of Clinical Oncology, Aichi Cancer Center Hospital

Department of Internal Medicine, Shikoku Cancer Center

(2) Collection of blood samples

A collaborator in each institution^*3^ collects 5 mL of blood in an EDTA 2Na-containing blood collection tube from a patient who is able to provide a blood sample. The blood samples are given the same institutional case numbers as the FFPE samples, personal data of the patients is encrypted, and the blood samples are collected by SRL. SRL extracts DNA from the blood samples, with each genomic DNA identified by an institutional case number. SRL sends the extracted DNA to the Center for Research Administration and Support of National Cancer Center (see the attached document, “Operation procedure of collection of samples”). From there, every genomic DNA with an institutional case number is delivered to a personal data manager at the National Cancer Center Hospital East, who replaces the institutional case numbers with new sample codes. Finally, after linkable anonymization, the genomic DNA with a sample code is delivered to the research office (see the attached document, “Flow chart of the study, No.2 [Blood sample]”).

*3: Handling of samples by the Shizuoka Cancer Center is organized as follows.

G&G SCIENCE CO., LTD., which is a principal corporate research participant in the study, establishes a laboratory, dispatches researchers within the Shizuoka Cancer Center Research Institute, and prepares a collaboration contract that covers also security issues. G&G SCIENCE CO., LTD. performs research, such as genetic analysis, in accordance with paragraph “7.4.5 Protection of privacy and handling of patient data.”

SRL sends HE-stained slides and remaining unstained slides, as well as extracted DNA, to a personal data manager at the Shizuoka Cancer Center, who replaces the institutional case number and assigns a new sample code, and after linkable anonymization, delivers the FFPE samples and genomic DNA with a sample code to the principal investigator at the Shizuoka Cancer Center (see the attached document, “Operation procedure of collection of samples.”).

**4.2.2 DNA extraction from FFPE samples^*4^**

The manager of sample/clinical data in the research office asks a genetic data manager to send the HE-stained slides and unstained slides associated with a sample code to the Exploratory Oncology Research & Clinical Trial Center, National Cancer Center, where genomic DNA is extracted from the FFPE samples.

Genomic DNA of cancerous tissue is extracted from colon cancer cells that are specifically dissected from FFPE samples with laser capture microdissection. To provide material for comparison, genomic DNA is extracted also from non-cancerous tissue. The quality of the extracted genomic DNA is checked in the way defined by each institution (purity, degree of DNA fragmentation, possibility of amplification). Only samples that are judged to provide highly reliable analysis results are used.

*4: Handling of samples by the Shizuoka Cancer Center is determined as follows.

Send HE-stained slides and unstained slides associated with a sample code to the Exploratory Oncology Research & Clinical Trial Center, National Cancer Center, from Shizuoka Cancer Center Research Institute. The genomic DNA is extracted from the FFPE samples at the Exploratory Oncology Research & Clinical Trial Center, National Cancer Center.

**4.2.3 Whole exon sequence analysis^*5^**

DNA is extracted from cancerous and non-cancerous tissues collected from all FFPE samples, as well as blood from all samples for which consent is obtained at the Exploratory Oncology Research & Clinical Trial Center, National Cancer Center. The whole exon region is enriched by the target capture method. The aim is to sequence 10 depths or more, covering ≥ 90% of the whole exon, using a high-speed sequencer, such as the Illumina Genome Analyzer. A fluorescent beads array system is used to identify exons with a genomic DNA mutation or structural anomaly.

The Exploratory Oncology Research & Clinical Trial Center, National Cancer Center, accepts collaborative researchers from G&G SCIENCE CO., LTD., who handle the whole exon sequence analysis.

**4.2.4 GWAS analysis/CNV analysis^*5^**

GWAS and CNV analyses using a SNP array are performed using the Illumina iScan System. DNA is extracted from cancerous and non-cancerous tissues of all FFPE samples, as well as blood from all samples from which consent is obtained. DNA fragmented by formalin fixation is assessed by real time PCR and repaired prior to SNP genotyping using the Infinium HD Assay. For FFPE samples, the aim is to obtain data for ≥ 90% of the call rate using Illumina’s HumanOmniExpress_FFPE-12, which is suitable for DNA extracted from formalin-fixed specimens. For more precise analysis, blood samples are used and the aim is to obtain data for ≥ 99% of the call rate using HumanOmniExpress-12.

*5: Handling at the Shizuoka Cancer Center is determined as follows.

DNA extracted from cancerous and non-cancerous tissues collected from all FFPE samples at the Exploratory Oncology Research & Clinical Trial Center, National Cancer Center, is sent to the Shizuoka Cancer Center Research Institute through the research office. DNA extracted from blood of all samples for which consent was obtained is delivered to the Shizuoka Cancer Center Research Institute through the principal investigator of the Shizuoka Cancer Center, and they are analyzed at the Shizuoka Cancer Center Research Institute.

However, when analyses cannot be conducted at the Shizuoka Cancer Center Research Institute, they are conducted at the Exploratory Oncology Research & Clinical Trial Center, National Cancer Center, and the National Center for Global Health and Medicine. Each analytical institution should exchange a memorandum with the Shizuoka Cancer Center in which they agree to sincerely respond to an audit by the Shizuoka Cancer Center.

Analyses and availability at the Shizuoka Cancer Center Research Institute at this time are shown below.

| Cohort | Analysis | Availability of analysis at the Shizuoka Cancer Center Research Institute |
| --- | --- | --- |
| For exploration | Whole exon sequencing | No |
|  | Fluorescent beads array | Yes |
|  | GWAS | No |
|  | CNV | No |

**4.2.5 Analysis of genetic data**

When genomic data obtained from GWAS or whole exon sequencing is analyzed, it is also tabulated considering the function of gene products. This data is used as a reference to identify gene mutations related to the therapeutic effect.

(1) Evaluate the presence or absence of somatic mutations in colon cancer and other types of cancer using the COSMIC database.

(2) Evaluate the general level of expression in cancer tissues, referring to a Gene Expression Omnibus such as Gene Logic or GEO.

(3) Estimate the impact (loss-of-function mutation, gain-of-function mutation) affecting protein function due to amino acid substitution induced by gene mutation, using the BLOSSUM matrix and databases such as Polyphen or Canpredict, and conduct weighting of mutations.

(4) Tabulate gene products with mutations and categorize them by their accumulated sites, such as functional groups (receptor tyrosine kinase, chromatin modifier) or intracellular signaling pathways (MAPK pathway, NF-κB pathway), using a functional annotation database, such as KEGG or GO. In order to add a gene product for which functional annotation is insufficient, we utilize a prediction algorithm such as DAVID.

**4.2.6 Patient background, collection of clinical efficacy data^*6^**

Register patient background and data on clinical efficacy data in the web-CRF system by extracting appropriate data from medical records and accompanying data.

**4.2.7 Case report form (CRF)^*6^**

The CRF is composed of (1) the Eligibility confirmation form, “Eligibility confirmation form for the exploratory cohort, Eligibility confirmation form for the inference cohort,” (2) Research on the details of patient background and data on efficacy and safety, “Pretreatment record form 1, Pretreatment record form 2, Pretreatment record form 3”, and (3) Additional research on efficacy and safety “Progress record form: RECIST Version 1.1, Prognosis survey form, Follow-up study form (which is performed regularly until March 31, 2016, only for patients who are undecided on any one of the following: safety, response rate, progression-free survival, and overall survival).”

**4.2.8 Therapy and safety evaluation**

Investigators in each institution conduct therapy evaluation in accordance with the RECIST guidelines (Version 1.1). A safety evaluation is conducted using CTCAE (Common Terminology Criteria for Adverse Events) Version 4.0, JCOG version translated in Japanese (CTCAE v4.0 - JCOG).

**4.2.9 Collection of clinical data^*6^**

Investigators in each institution confirm the eligibility of study patients and enroll them using the web enrollment system. Only clinical data should be entered in the attached documents: web-CRF(1) Eligibility confirmation form, web-CRF(2) Research on details of patient background and data on efficacy and safety, and web-CRF(3) Additional research on efficacy and safety. Besides the web enrollment system, paper CRFs are filled in and faxed to the Clinical Trial Support Office, National Cancer Hospital East. To avoid the risk of leaking patient personal data, an original institutional case number should be used in place of the chart numbers of each institution.

The Center for Research Administration and Support of the National Cancer Center verifies that no clinical data data associated with the institutional case number have been deleted. If there is a deletion, the form is returned to a collaborator in each institution before ordering linkable anonymization to a personal data manager. If there is no deletion, the clinical data associated with the institutional case number is delivered to the personal data manager. The personal data manager deletes the institutional case number and assigns the same sample code as that of the corresponding sample. After linkable anonymization, the clinical data associated with the sample code is delivered to the research office. Database preparation and data management are carried out at the Center for Research Administration and Support of the National Cancer Center.

Additional research on efficacy and safety is performed regularly (until March 31, 2016) only for patients who are undecided on any of the following: safety, response rate, progression-free survival, and overall survival, using web-CRF(3) Additional research on efficacy and safety. Linkable anonymization is similarly conducted as described in the above procedure.

The web enrollment URL is separately notified by the “Web enrollment system using manual.” (Enrollment is available for 24 hours; when not available due to maintenance or other causes, users are informed beforehand.)

*6: Handling of clinical data by Shizuoka Cancer Center is determined as follows.

A researcher in the Shizuoka Cancer Center confirms the eligibility of study patients and fills in clinical data associated with the institutional case number in the attached documents: CRF(1) Eligibility confirmation form, CRF(2) Research on details of patient background and data on efficacy and safety, and CRF(3) Additional research on efficacy and safety. The researcher delivers them to the personal data manager at the Shizuoka Cancer Center, who replaces the institutional case number with the same sample code as that of the corresponding sample. After linkable anonymization, the clinical data associated with a sample code is delivered to the principal investigator at the Shizuoka Cancer Center, who sends it to the research office at the National Cancer Center by FAX or mail. Database preparation and data management are conducted at the Center for Research Administration and Support of National Cancer Center. To avoid the risk of leaking patient personal data, use of a chart number should be avoided.

Additional research on efficacy and safety is performed regularly (until March 31, 2016) only for patients who are undecided on one of the following: safety, response rate, progression-free survival, and overall survival, using CRF(3) Additional research on efficacy and safety. Linkable anonymization is similarly conducted as described in the above procedure.

Contact address for web enrollment

Center for Research Administration and Support of National Cancer Center

From 10 am. to 5 pm. on weekdays (Closed on Saturdays, Sundays, and public holidays)

**4.3 Analysis of the inference cohort^*7^**

Similar procedures are followed as for the exploratory cohort.

• Samples and their collection → See 4.2.1

• DNA extraction from FFPE samples → See 4.2.2

• Patient background, collection of clinical efficacy → See 4.2.6

• Case report form (CRF) → See 4.2.7

• Evaluation of therapeutic effect and safety → See 4.2.8

• Collection of clinical data → See 4.2.9

**4.3.1 Gene mutation/polymorphism analysis^*7^**

DNA extracted from cancerous and non-cancerous tissues collected from FFPE samples, or DNA extracted from blood, are used to search for genomic mutations, polymorphisms, or a region with a structural abnormality. This is accomplished through analysis of the exploratory cohort by target resequencing or fluorescent beads array.

*7: Handling of samples by the Shizuoka Cancer Center is completed as follows.

DNA extracted from cancerous and non-cancerous tissues collected from FFPE samples at the Exploratory Oncology Research & Clinical Trial Center, National Cancer Center, is sent to the Shizuoka Cancer Center through the research office. DNA extracted from the blood of all samples for which consent was obtained is delivered to the Shizuoka Cancer Center Research Institute through the principal investigator at the Shizuoka Cancer Center. All DNA is analyzed at the Shizuoka Cancer Center Research Institute.

However, when analyses cannot be conducted at the Shizuoka Cancer Center Research Institute, they are conducted at the Exploratory Oncology Research & Clinical Trial Center, National Cancer Center, and the National Center for Global Health and Medicine. Each analytical institution should exchange a memorandum with the Shizuoka Cancer Center in which they agree to sincerely respond to audit by the Shizuoka Cancer Center.

Analyses and availability at Shizuoka Cancer Center are shown below.

| Cohort | Analysis | Availability of analysis at Shizuoka Cancer Center Research Institute |
| --- | --- | --- |
| For inference cohort | Target resequencing | Yes |
|  | Fluorescent beads array | Yes |

**4.4 Correlating DNA analysis with clinical data**

See 6. Statistical analysis.

**4.5 Flow of samples and genetic data^*8^**

**See the attached documents “Flow chart of the study 1, 2.”**

[1] If consent can be obtained from a patient, obtain consent, enroll the patient, and enter the clinical data and the institutional case number in the web-CRF system. If consent cannot be obtained from a patient, enroll the patient and enter the clinical data and the institutional case number in the web-CRF system.

Each institution ([1], [2], [3])

A

Encryption

[2] Materials required for the samples are prepared and sent by MEDICAL & BIOLOGICAL LABORATORIES CO., LTD. At each institution, predefined FFPE samples (and blood samples in some cases) should be prepared, and a label with the institutional case number should be put on each slide and blood collection tube.

[3] Fill in the encryption specific request form by SRL (4 copies), including the chart number and institutional case number, and store the first and second sheets (for institutions 1 and 2, respectively).

[4] SRL collects the above-described FFPE samples, blood samples and encryption specific request form (the third and fourth sheets) and then encrypts the samples (chart numbers are not copied on the third and the fourth sheets). [Flow chart of the study: Arrow A]

SRL, Inc. ([4], [5])

B

[5] HE staining of slides of cancerous and non-cancerous tissues from FFPE samples is conducted at SRL. When a blood sample is available, genomic DNA should be extracted. SRL sends the samples associated with the institutional case number to the Center for Research Administration and Support of National Cancer Center. [Flow chart of the study: Arrow B]

[6] The HE-stained slides and remaining unstained slides associated with the institutional case number, and DNA extracted from blood are sent from the Center for Research Administration and Support of National Cancer Center to a personal data manager at the National Cancer Center Hospital East. [Flow chart of the study: Arrow C]

C

Center for Research Administration and Support of National Cancer Center [6]

[7] The personal data manager replaces the institutional case number with a sample code (linkable anonymization). The correspondence table between an institutional case number and a sample code is strictly controlled by the personal data manager. The personal data manager delivers the HE-stained slides and remaining unstained slides associated with a sample code, and DNA extracted from blood to a sample/clinical data manager at the research office of the National Cancer Center. [Flow chart of the study: Arrow D]

D

Manager of personal data [7]

Linkable anonymization

Storage of the correspondence table

[8] The sample/clinical data manager at the research office of National Cancer Center sends the HE-stained slides and unstained slides associated with a sample code to the Exploratory Oncology Research & Clinical Trial Center, National Cancer Center, through a genetic data manager. [Flow chart of the study: Arrow E]

National Cancer Center

Exploratory Oncology Research & Clinical Trial Center [9]

E

Research office [8]

A manager of data/sample

[9] Genomic DNA is extracted from the slides at the Exploratory Oncology Research & Clinical Trial Center, National Cancer Center, and is sent to the research office. [Flow chart of the study: Arrow F]

[10] The genetic data manager at the research office sends genomic DNA associated with a sample code for genetic analysis to participating institutions (Exploratory Oncology Research & Clinical Trial Center, National Cancer Center, National Center for Global Health and Medicine, and G&G SCIENCE CO., LTD). [Flow chart of the study: Arrow G]

G

F

Research office
([10], [11])

[11] The genetic data manager at the research office prepares archives and stores remaining samples in a predefined storage area.

Genetic analysis institutions [12]

[12] Genetic analysis of the genomic DNA associated with a sample code is performed at the participating institutions. Remaining genomic DNA and genomic data associated with a sample code are returned to the research office. [Flow chart of the study: Arrow H]

H

[13] The research office integrally analyzes genomic and clinical data associated with a sample code.

Research office [13]

The attached documents [Flow chart of the study: Arrow I, Arrow J] indicate that the CRF (clinical data and data about the institutional case number) was sent to the personal data manager from each institution through the Center for Research Administration and Support of National Cancer Center. [Flow chart of the study: Arrow K] indicates that the CRF (clinical data and data about sample codes) was sent from the personal data manager to the research office. The Center for Research Administration and Support of National Cancer Center verifies that no clinical data data associated with the institutional case number have been deleted. If there is a deletion, return it to a collaborator of each institution before ordering linkable anonymization to the personal data manager. If there is no deletion, deliver the clinical data associated with the institutional case number to the personal data manager. The personal data manager replaces the institutional case number with the same sample code corresponding to the sample, and after linkable anonymization, delivers the clinical data associated with a sample code to the research office.

*8: See the attached documents “Flow chart of the study 1, 2, Shizuoka Cancer Center” for handling at the Shizuoka Cancer Center.

**4.6 Flow of anonymization^*9^**

|  | Collaborator of each institution | SRL | Center for Research Administration and Support of National Cancer Center | Manager of personal data | Research office | Genetic analysis institution | Manager of typing/statistical analysis |
| --- | --- | --- | --- | --- | --- | --- | --- |
| Patient ID | ○ |  |  |  |  |  |  |
| Clinical data associated with an institutional case number | ○ | ○ | ○ | ○ |  |  |  |
| Sample associated with an institutional case number | ○ | ○ | ○ | ○ |  |  |  |
| Clinical data associated with a sample code |  |  |  | ○ | ○ |  | ○ |
| Genomic sample associated with a sample code/data |  |  |  | ○ | ○ | ○ | ○ |

*9: Handling at Shizuoka Cancer Center (SCC) is determined as follows.

|  | Researcher in SCC | SRL | Manager of personal data in SCC | Principal investigator in SCC | Research office | Genetic analysis institution | Manager of typing/statistical analysis |
| --- | --- | --- | --- | --- | --- | --- | --- |
| Patient ID | ○ |  |  |  |  |  |  |
| Clinical data associated with an institutional case number | ○ | ○ | ○ |  |  |  |  |
| Sample associated with an institutional case number | ○ | ○ | ○ |  |  |  |  |
| Clinical data associated with a sample code |  |  | ○ | ○ | ○ |  | ○ |
| Genomic sample associated with a sample code/data |  |  | ○ | ○ | ○ | ○ | ○ |

**Genetic analysis institutions**

Exploratory Oncology Research & Clinical Trial Center, National Cancer Center (G&G SCIENCE CO., LTD. dispatches a researcher)

National Center for Global Health and Medicine (G&G SCIENCE CO., LTD. dispatches a researcher)

Shizuoka Cancer Center Research Institute^*10^ (G&G SCIENCE CO., LTD. formally establishes a laboratory and dispatches a researcher)

*10: Samples from the Shizuoka Cancer Center are analyzed at the Shizuoka Cancer Center Research Institute. As for samples which are difficult to be analyzed there, analysis is carried out at the Exploratory Oncology Research & Clinical Trial Center, National Cancer Center, and the National Center for Global Health and Medicine. Each analytical institution should exchange a memorandum with the Shizuoka Cancer Center in which they agree to sincerely respond to an audit by the Shizuoka Cancer Center.

**5. Target sample size and research period**

**5.1 Target sample size**

[Exploratory cohort sample]: Maximum 150 cases.

Fifty cases of the super-responder group (maximum), 50 cases of the no response group (maximum), and 50 cases that developed serious adverse events (maximum) among those which satisfy the criteria and received therapy with medications including cetuximab, between September 2008 and May 2010.

[Inference cohort sample]: 250 or more cases.

Accumulate 250 or more cases which satisfied the eligibility criteria and received therapy with medications including cetuximab or panitumumab, between June 2010 and the date of approval by the IRB at each institution.

**5.2 Research period**

From the date of approval by the IRB until March 31, 2016.

**6. Statistical analysis**

The main purpose of the study is to identify a new genetic biomarker that predicts clinical efficacy of the anti-EGFR antibody. The study consists of two steps: (1) a phase whereby target gene groups are identified (6.1 Analysis of the exploratory cohort), followed by (2) a phase in which statistical inference on the predictability of extracted gene groups is performed (6.2 Analysis of the inference cohort). Although the details of each phase are determined separately in the statistical analysis plan prior to the analysis, a rough policy is defined here below. Statistical analysis is conducted at the research office of the National Cancer Center Hospital East.

**6.1 Analysis of the exploratory cohort**

The whole exon sequence is analyzed while exploring gene groups. Generally, a method which divides the average patient population into a training set and a test set is considered. That is, after obtaining genomic data from the training set, candidate gene groups are extracted from it and a statistic model is constructed (model parameters are estimated by applying them to an appropriate parametric model) to confirm the appropriateness of the obtained model in an independent test set. This procedure was initially considered also for the study.

However, an analysis of the whole exon sequence, which is a feature of the study, requires a very high cost per sample. Therefore, a realistic analysis of the exploratory phase has to be completed using as few samples as possible. Based on this, and the need to extract a gene with strong correlation to the clinical efficacy of the anti-EGFR antibody as fast as possible, we decided to adopt the following analytical procedure, after conferring with researchers from various specializations.

(1) The participating institutions are leaders in clinical achievement in Japan, and they have accumulated clinical data of wild-type cases treated after the approval of cetuximab in 2008. Thus, these data should be used in the exploratory phase. First, cases should be classified either as super-responders with high clinical efficacy, or as no response cases, which show no efficacy. Each institution is then allowed to accumulate a sufficient number of cases that meet the conditions. For the definitions of a super-responder and a non-responder, the following amended version is used based on RECIST guidelines.

Super-responder: PR/CR or PFS ≥ 6 months according to RECIST guidelines (Version 1.1)

Non-responder: Cases for which initial evaluation was PD, although its drug intensity was retained at 80% or more according to RECIST guidelines (Ver 1.1)

(2) N cases of complete response cases are selected as super-responder (SR) and N cases of no response cases are selected as super non-responder (SNR) from all accumulated cases (the value N is described later). Here, the definition of SR and SNR is a problem. To solve the problem, prior to the study, the study group conducted a pilot study in which SR/SNR was ranked in subjects who were enrolled in the previously conducted Study GI-biomarker0902 (Multicenter, retrospective study on the clinical efficacy of cetuximab therapy in unresectable recurrent colon cancer with or without *KRAS* gene mutation). As a result, the generalized kappa coefficient^18)^ was found to be extremely high by medical oncologists (as the results are undisclosed, the details are omitted). Therefore, we determined that SR/SNR could be defined in the same manner by medical oncologists in this study as well.

(3) After selecting N cases of SR and N cases of SNR, we evaluate the correlation between the data of the therapeutic effect of the 2N cases and the mutation data of each gene obtained from analysis of the whole exon sequence, and extract a candidate gene group. Methods to adjust the multiplicity of a test, the family-wise error rate (FWER; a type I error rate), or the false discovery rate (FDR)^19)^ are considered. Generally, since a method of regulating the FWER is conservative in a genome-wide analysis, a method to adjust the FDR is often used when reducing statistical power. Therefore, the FDR is adopted here.

(4) Although candidate gene groups are extracted by referring to the value of the FDR, since there is no similar preceding data using the whole exon sequence for the prediction of clinical efficacy of anti-EGFR antibodies at this time, it is currently unclear what kind of data will be obtained. For this reason, although the threshold on the FDR is used as the standard, ultimately several to a few dozen candidate genes will be extracted, using the size of the odds ratio and known biological data as required.

(5) As described above, analysis of the exploratory phase is requested to be completed with as few samples as possible. For this reason, first a total of 50 cases (super-responder: N = 25, non-responder: N = 25) are analyzed. If it is considered that clinically/biologically/statistically sufficient results are obtained at this phase, it is possible to move to the next phase described below. Otherwise, another 50 cases (super-responder: N = 50, non-responder: N = 50) should be added, for a total of 100 cases. More than 100 cases cannot be added due to budgetary and sample size constraints.

(6) To explore a candidate gene that predicts serious adverse events, GWAS analysis is also conducted in the exploratory cohort. For GWAS analysis, 25 cases that developed serious adverse events should first be identified, and then analyzed together with the previously described 50 cases of super-responder and 50 cases of non-responder as a control. If a promising result is obtained at this step, it is possible to move to the next phase; however, if not, another 25 cases should be added for a total of 150 cases.

**6.2 Analysis of the Inference cohort**

In the exploratory cohort, in order to conduct the whole exon sequencing analysis, which is a promising but costly method, case groups with remarkable or non-clinical efficacy are selected. A similar approach of dividing into 2 polarized groups based on clinical efficacy has been used before in a report by Mizokami et al.^11)^ However, because the exploratory cohort diverges from the original subjects, it is unclear to what extent the extracted candidate genes can make a prediction about the average patient population. Therefore, a cohort in which statistical inference will be conducted on extracted genes from the exploratory cohort is independently prepared.

Specifically, 250 patients who received treatment with an anti-EGFR antibody between June 2010 and the date of approval by the ethical review board of each institution, are enrolled. Thus, the genes extracted from the exploratory cohort are evaluated in the inference cohort. The analytical policy is as follows.

(1) $K$-fold cross-validation ($K$-fold CV) is conducted in 250 or more patients enrolled in the inference cohort. Of the patients that are randomly divided by cross-validation, a set that comprises 150 × $\left(1/K\right)$ subjects is represented as $V_{k}$, and the rest of the training set that comprises 150 × $\left(1-1/K\right)$ is represented as $T_{k}$. The voting method, a sort of machine learning algorithm, is applied to the set $T_{k}, k=1, \ldots, K,$.

(2) The number of candidate genes extracted from the exploratory cohort is determined as $G$. Apply the proportional hazard model to each $G$ gene (univariate) in the set $T_{k}, k=1, \ldots, K,$ and speculate the hazard ratio on a progression-free survival (PFS). In the set $V_{k}$, classify the patients that have $L_{k}$ or more gene mutations for which the hazard ratio is more than the threshold $R_{k}$ speculated in $T_{k}$ as “sensitive” and the patients that have less than $L_{k}$ gene mutations as “non-sensitive.” That is, when they have $L_{k}$ or more gene mutations of

$$\exp(\hat{\beta}_{j})\geq R_{k}$$

they are classified as sensitive patients. According to this operation, patients in the set $V_{k}$ are labeled as either a sensitive example $S_{k}$ or a non-sensitive example $V_{k}-S_{k}$. In practice, since the best pair $(R_{k}, L_{k})$ should be determined in the set $T_{k}$, determine the pair by conducting a (nested) $K$-fold cross validation in the set $T_{k}$. Repeat the above operation for all $k=1, \ldots, K$.

(3) Since 250 or more patients enrolled in the inference cohort are classified into either a sensitive group or a non-sensitive group $S=\cup_{k=1}^{K}S_{k}$, calculate the *P* value on the hazard ratio of the two groups based on the permutation test. Candidate genes extracted from the exploration cohort are evaluated as above.

(4) Since the patients from the exploratory cohort are all administered with anti-EGFR antibodies, in a strict sense it is hard to say that the evaluation could predict the effect of anti-EGFR antibodies. In addition, patients belong to restricted institutions even though they are representative of institutions in Japan. Therefore, even if encouraging results are obtained by statistically appropriate speculation in the inference cohort, it cannot be denied that generalizability (external validity) of the extracted genes is uncertain. For these reasons, we are going to conduct true validation by utilizing archived specimens obtained from a recently reported or a future phase III clinical trial (multinational study), taking into account inclusion of subjects who did not receive treatment with the anti-EGFR antibody.

(5) We also construct a similar prediction model using cross-validation for candidate genes on adverse events obtained from the GWAS analysis in the exploration cohort. When the incidence of adverse events is low, we consider development of an appropriate prediction model.

**7. Ethical issues**

**7.1 Protection of patients**

**7.1.1 Protection of patients by anonymization**

Patients are protected through linkable anonymization, which is performed by a personal data manager at the National Cancer Center Hospital East.^*11^ Samples and clinical data are anonymized, and data are stored in a specific computer associated with a login password. Only the personal data manager can conduct the matching.

*11: Clinical data and samples in the Shizuoka Cancer Center are linkable anonymized by a personal data manager at the Shizuoka Cancer Center.

**7.1.2. Expected benefits of participating in the study**

There is no direct gain for patients who participate in the study. However, patients who are scheduled to receive cetuximab or panitumumab may benefit from acquiring knowledge on the therapeutic effect and risks of adverse drug reactions, which may prove beneficial when receiving therapy in the future.

**7.1.3 Predicted risks and disadvantages for subjects of the study**

Since the study uses previously collected tissue samples, it is free of disadvantages associated with tissue collection. However, 5 mL of blood is newly collected from a patient from whom a consent on blood collection is obtained. Possible disadvantages include pain and/or subcutaneous hemorrhage at the blood collection site.

**7.1.4. Delivery of samples to outside institutions and the contract**

When samples or genomic data are supplied to an outside institution, the correspondence table derived from linkable anonymization should not be provided. In addition, when duties pertain to treatment/storage/managing the system of samples, or construction of the database, the contents of safety management measures relating to personal data should be explicitly provided.

**7.2 Ethical guidelines**

The study complies with the Declaration of Helsinki and the Ethical Guidelines for Human Genome/Gene Analysis Research, which is a joint guideline by 3 government ministries. Protection of human rights of the subjects and matters concerning patients' agreement follow the rules approved by the IRB. If the Declaration of Helsinki and Ethical Guidelines for Human Genome/Gene Analysis Research are amended, the study is to be conducted in accordance with the amended contents.

**7.3 Approval by the institutional review board**

The study is conducted after the protocol and the informed consent form are evaluated and approved by the IRB of each institution (see the attached document “Written Informed Consent form/Withdrawal of consent”).

**7.4 Basic attitude toward patients**

**7.4.1 Handling a person from whom informed consent can be obtained**

If consent from the patient or the patient's legally acceptable representative can be obtained, an agreement for use of FFPE samples that are collected before the date of approval by each IRB should also be obtained. The investigator should take into account the ethical considerations for conducting the study and obtaining a written agreement from the patient, explaining them the following matters from (1) to (22) using the written informed consent form approved by the IRB from each institution. Typically, consent should be obtained from the patient; however, if communication is extremely difficult, consent from the patient's legally acceptable representative is accepted. The term “legally acceptable representative” used in the study means the subject's spouse or guardian, or any other person who is considered such a representative in view of both parties' actual life and mental partnership and who speaks for the subject’s best interests.

(1) About the written informed consent form.

(2) About genes.

(3) About purpose of the study.

(4) About the reason we ask you to participate in the study.

(5) About samples.

(6) About policy on protection of genetic data.

(7) About samples and data after genetic analysis.

(8) About results of the genetic analysis.

(9) About planned participation period and number of participants.

(10) About potential benefits and risks in participation in the genetic analysis.

(11) About that your participation is on a voluntary basis and you can withdraw from the study at any time you want.

(12) About compensation and treatment.

(13) About what happens when new data is obtained.

(14) About ethical review of the study.

(15) About protection of personal data.

(16) About research funding and conflicts of interest.

(17) About expectations for participants during the study period.

(18) About attribution of intellectual property rights.

(19) About genetic counseling for anxiety before and after genetic analysis.

(20) About principal investigator of the multicenter study.

(21) About that you may ask questions at any time you want.

(22) About contact address.

In addition, written consent is obtained for each item, such as provision of the blood sample, secondary uses of samples and data in future medical research, and disclosure of data if genetic evidence related to a disease is found.

Before obtaining their consent, patients should be provided with sufficient time and opportunities to ask questions and receive satisfactory answers, to determine whether they wish to participate in the study or not. The investigator who provides explanations signs the consent form and enters the date. The original consent form should be appropriately stored, such as attached to the chart.

**7.4.2 Handling of a person from whom informed consent is difficult to obtain**

When the patient has died, there is trouble in contacting the patient, or consent from the patient or the patient's legally acceptable representative is impossible or difficult to obtain, handle the samples according to Group C human specimen guidelines. These are described in the above joint guideline by 3 government ministries and refer to a human specimen for which consent to use it in research has not been given at the time of donation. In this case, approval by the IRB and permission from the head of the research institution are required. The study satisfies the requirements specified by the joint guideline to use Group C human specimens after linkable anonymization, based on the facts described below (double underlined parts). It should be confirmed before enrollment that each case satisfies all of the requirements described below (from [1] to [5]) by CRF.

(1) Possible risks and disadvantages for patients by Human Genome/Gene Analysis Research are considered to be extremely low.

• The samples handled in the study are used for analysis after being anonymized. Researchers do not have the correspondence table for previously collected specimens to protect personal data; therefore, the possible risks or disadvantages for patients are extremely low.

(2) Human Genome/Gene Analysis Research is for the improvement of public health.

• The study is highly likely to identify biomarkers related to the efficacy of drugs or adverse drug reactions, and these biomarkers will lead to improvements in public health in the form of new diagnostic and therapeutic agents.

(3) Human Genome/Gene Analysis Research is virtually impossible by other methods.

• The study can only be conducted by analyzing samples with reliable clinical data. If the samples of the study are collected by obtaining approval in a prospective clinical trial, the case enrollment period and the observation period are considered to be at least 5 years; it means operation of the study is virtually impossible. The institutions that participated in the study have already had a 500-case scale cohort associated with reliable clinical data, and the study can be conducted by utilizing the samples that satisfy the conditions of use, such as for Group C human specimens.

(4) Any data on the state of operation of Human Genome/Gene Analysis Research should be disclosed. Measures should be taken for patients and the patient’s legally acceptable representative to refuse inquiries and use of samples for research.

• Data on the state of operation of the study is disclosed on the website of each institution which participates in the study, including the National Cancer Center. The contact address for the patients or the patient’s legally acceptable representative should be specified and inquiries should be answered. Samples of patients who refuse their use for research are not to be used. In addition, if a patient's sample and data have been sent to the research office before their decision to refuse is indicated, discard the sample and data stored at the research office and exclude them from analyses after that.
(See the attached documents “Contents of the open website")

(5) Obtaining consent from the provider or the provider's legally acceptable representative is difficult

• When samples such as Group C human specimens are used in the study, they are limited to cases where the patient was dead, or obtaining consent is impossible or difficult.

In the former comprehensive agreement of the National Cancer Center, when a patient refuses to allow his/her samples to be used for research at the time of provision, the samples shall not be used, respecting the patient's own will.

**7.4.3 Disclosure of genetic data**

Genetic data should not be disclosed to the patient in person. If a patient hopes his/her genetic data to be disclosed in written form, it should be done so in person. However, if he/she wants disclosure of the entire or most of the genomic analysis data, the risks of distributing detailed genomic data to society should be explained to him/her. When it is assumed that life, body, assets, and other rights and interests of the patient or a third party may be harmed by providing the genetic data, no part of it is to be disclosed.

When gene mutations for which a relationship with a disease has been determined are found in the course of this study, the patient should be notified in person only when it is certain that the genetic data might affect the lives of the patient and blood relatives and there is an effective way of dealing with it. When the patient is notified, disclosure should occur under close cooperation with the investigator, giving adequate consideration to its medical or mental implications. Genetic counseling should be offered, if needed.

When the data might affect the lives of the provider and blood relatives and there is an effective way of dealing with it, but the patient nevertheless does not want the genetic data to be disclosed, approval from the IRB at each institution is required. In this case, the principal investigator in each institution will ask the patient in person again whether he/she wants to disclose it or not. When the patient wants disclosure, this should be done in close cooperation with the investigator, giving adequate consideration to its medical or mental implications. Genetic counseling should be offered, if needed.

The contact person for disclosure of data to the patient in person is typically the principal investigator at each institution.

**7.4.4 Genetic counseling**

When genetic counseling is needed, this should be dealt in the appropriate way with a genetic counselor from each institution or an outpatient department of genetic counseling at the National Cancer Center.

**7.4.5 Protection of privacy and handling of patient data**

All researchers and persons concerned with the study have a duty of confidentiality as data handlers, and shall make the best effort to protect personal data and privacy.

The correspondence of chart numbers kept at each institution with institutional case numbers from the study is encrypted by the time SRL collects samples. Thus personal data shall never be leaked to outside institutions. When the clinical data from each institution is provided to a personal data manager through the Center for Research Administration and Support of National Cancer Center, an institutional case number is provided. The personal data manager at the National Cancer Center Hospital East prepares the correspondence table between an institutional case number and a sample code, and anonymizes it by replacing the institutional case number of the sample with the new sample code. The personal data manager delivers samples associated with a sample code only to the research office. When samples are provided to the genetic analysis institutions from the research office, only the sample codes, not the correspondence table, are given. The genetic analysis institution reports sample codes and analyzed genomic data to the research office, where statistical and data analysis is performed.

Since any personal identifier is given by a personal data manager to the research office and the genetic analysis institution after conversion to a sample code by encryption and linkable anonymization, genomic data and personal data will never be directly linked.

The genetic analysis institution accepts researchers from a collaborative institution, G&G SCIENCE CO., LTD., who handle the whole exon sequence analysis. However, at this point the data have been already anonymized. The data of the whole exon is analyzed at the Exploratory Oncology Research & Clinical Trial Center, National Cancer Center, and the data is not shared with G&G SCIENCE CO., LTD.

**7.5 Methods for storing and discarding samples^*12^**

In principle, samples including DNA are stored at the research office under anonymization and discarded after completion of the study. DNA extracted from clinical samples is discarded as medical waste after the label of the sample code is completely deleted and the DNA is destroyed by sodium hypochlorite. However, in the cases whereby agreement was obtained for “An item to inquire an agreement on secondary use”, samples are stored at the research office for 5 years after the completion of the study for future medical research. When stored samples are used in future medical research, the principal investigator, the research office, or a responsible researcher designated by them shall prepare a new protocol and receive approval from each IRB as to which samples approved for secondary use were submitted after evaluation.

*12: Handling of samples by the Shizuoka Cancer Center is as follows.

Samples, including DNA, are stored at the Shizuoka Cancer Center Research Institute under anonymization. In principle, the storage period of samples should be 10 years after completion of the research, and the samples are discarded after the storage period. When a sample is discarded, a report on the disposal of the sample is submitted. DNA extracted from clinical samples is discarded as medical waste after the label of the sample code is completely deleted and the DNA is destroyed by sodium hypochlorite. Secondary use of the stored samples is restricted to research associated with the study. The principal investigator, the research office, or a responsible researcher designated by them shall prepare a new protocol for the associated research and receive approval from the IRB of the National Cancer Center and the Shizuoka Cancer Center.

However, when the analysis cannot be conducted at the Shizuoka Cancer Center Research Institute, it is carried out at the Exploratory Oncology Research & Clinical Trial Center, National Cancer Center, and the National Center for Global Health and Medicine. Samples from the Shizuoka Cancer Center Research Institute are treated in the same way at each analytical institution. Each analytical institution should exchange a memorandum with the Shizuoka Cancer Center in which they agree to sincerely respond to an audit by the Shizuoka Cancer Center.

Managers of sample storage in each institution are as follows.

Exploratory Oncology Research & Clinical Trial Center, National Cancer Center

Katsuya Tsuchihara

National Center for Global Health and Medicine 　　　Masaya Sugiyama

Drug Discovery and Development Division, Shizuoka Cancer Center, Manager,

Masatoshi Kusuhara

**8. Presentation of the results of the research**

The principal investigator or a collaborator will publish the results of the study in scientific papers or at academic meetings. Co-authorship is restricted to persons who reviewed and agreed with the contents of the publication before submission.

**9. Secondary use of the data**

The data obtained from the study may be used secondarily only when approved by the investigator, in a way that is not linked to any personal identifiers (disclosure of the database or meta-analysis). When the primary data of the whole exon sequence is registered to the control access database, it must be approved by the IRB again.

**10.** **Compliance with the protocol**

The principal investigator at each institution and each staff member who participates in the study shall comply with the protocol to ensure the safety and human rights of the patients.

**11. Approval by the institutional review board**

**11.1 Approval at the beginning of the study**

When participating in the study, the protocol and the written data must be approved by an IRB from each institution and the head of the institution.

If approval by the IRB is obtained, a physician in each institution sends the documents approved by the IRB and a copy of the documents approved by the head of the institution to the research office. When the approved documents are directly delivered to the applicant (the principal investigator at the institution) by the IRB, only the IRB-approved documents are needed. The original approved documents are stored at the institution and a copy is stored at the research office.

Although written data to patients, which differs for each institution, can be used after being approved by the IRB from each institution, the contents in the protocol must not be changed. When a modification to the body of the protocol is requested by an IRB, the reasons why it should be amended or revised should be discussed with the research office. When the written data is modified by instruction of an IRB, the modified written data should be sent to the research office. The investigator/research office can ask for a revision to an IRB through the principal investigator of the institution when the modification is determined as inappropriate (deletion or modification of the contents).

**11.2 Annual update of approval at each institution**

The presence or absence of an annual update of approval of the protocol and the written information by the IRB follows the regulations of each institution participating in the study.

**11.3 Modification of the CRF**

When an imperfection, such as deletion of data required for the CRF, or an inappropriate category classification, is discovered after the start of the study, the CRF should be modified with the agreement of the investigator/research office. This can be done as long as it does not increase the medical/economic burden on enrolled patients. Modification of a CRF with no requirement to revise the body of the protocol is not defined as a revision of the protocol. A report on the modification of a CRF to the IRB of the institution, or the presence or absence of a revised application shall follow the regulations of the institution.

**12. Record keeping**

The research office takes on full responsibility for managing the data of anonymized CRF and the genomic data attached with a sample code.

**13. Report on discontinuation/completion of the clinical study**

When the clinical study is discontinued or completed, an outline of the results should be promptly provided to the head of the institution.

**14. Research funds and conflicts of interest**

The study is supported by research and development costs of Program 23-A-2 “Research for bridging base and clinical research, promotion of translational research to implement personalized medicine” from the National Cancer Center in the fiscal year 2011; Japan Science and Technology Agency’s A-STEP (Adaptable and Seamless Technology Transfer Program through Target-driven R&D), practical application challenge type “Development of small- and medium-sized enterprises/venture businesses;” and research funds provided by G&G SCIENCE CO., LTD. and MEDICAL & BIOLOGICAL LABORATORIES CO., LTD. Japan Science and Technology Agency’s A-STEP (Adaptable and Seamless Technology Transfer Program through Target-driven R&D), practical application challenge type “Development of small- and medium-sized enterprises/venture businesses” is commissioned to G&G SCIENCE CO., LTD. from Japan Science and Technology Agency, therefore, management of the research funding and research progress reports are operated by G&G SCIENCE CO., LTD. A part of the research effort is recommissioned from G&G SCIENCE CO., LTD. to each institution and funds are provided. MEDICAL & BIOLOGICAL LABORATORIES CO., LTD. is in charge of the administrative aspects of running a workshop, preparation of material for research, and contact points. No conflict of interest is declared in relation to this study. Any intellectual property that may arise from the study is managed under a separate collaborative research agreement.

**15.　Research organizations and researchers**

**Principal Investigator**

Atsushi Ohtsu

　Director of Exploratory Oncology Research and Clinical Trial Center, National Cancer Center

Yukiko Abe

　President of G&G SCIENCE CO., LTD.

**Research office**

Katsuya Tsuchihara

Division of Translational Research, Exploratory Oncology Research and Clinical Trial Center, National Cancer Center

Takayuki Yoshino

Department of Gastroenterology and Gastrointestinal Oncology, National Cancer Center Hospital East

Izumi　Miki

　National Cancer Center for Research Administration and Support

**Collaborator**

Hiroyasu　Esumi

Exploratory Oncology Research & Clinical Trial Center, National Cancer Center

Satoshi Fujii

　National Cancer Center Hospital East

Sachiyo Mimaki

　Exploratory Oncology Research and Clinical Trial Center, National Cancer Center

Satoshi Yuki

　Hokkaido University Hospital

Kentaro Yamazaki

　Shizuoka Cancer Center

Masatoshi Kusuhara

　Shizuoka Cancer Center

Yasuhiro Koh

　Wakayama Medical University

Eiji Shinozaki

　Cancer Institute Hospital of Japanese Foundation of Cancer and Research

Kiwamu Akagi

　Saitama Cancer Center

Kensei Yamaguchi

　Saitama Cancer Center

Kei Muro

　Aichi Cancer Center Hospital

Kohei Shitara

　Exploratory Oncology Research and Clinical Trial Center, National Cancer Center

Hideaki Bando

　National Cancer Center Hospital East

Tomohiro Nishina

　National Hospital Organization of Shilkoku Cancer Center

Takeharu Yamanaka

　National Cancer Center

Shogo Nomura

　National Cancer Center

Masashi Mizokami

　National Center for Global Health and Medicine

Nao Nishida

　National Center for Global Health and Medicine

Masaya Sugiyama

　National Center for Global Health and Medicine

Sumio Sugano

　The University of Tokyo

Yuzuru Suzuki

　The University of Tokyo

Takuya Koshizaka

　MEDICAL & BIOLOGICAL LABORATORIES CO., LTD.

Akiko Nakayama

　National Cancer Center

Noriko Kato

　National Cancer Center

**16.　Reference**

1) U.S. Department of Health and Human Services　Food and Drug Administration：Draft Guidance for Industry and Food and Drug Administration Staff - In Vitro Companion Diagnostic Devices. July 14, 2011

2) PFSB/ELD Notification 0120 No.1, Notification 0120 No.1 issued by the Safety Division, Pharmaceutical and Food Safety Bureau. January 20, 2011

3) Van Cutsem E., et al. *KRAS* status and efficacy in the first-line treatment of patients with metastatic colorectal cancer (mCRC) treated with FOLFIRI with or without cetuximab: The CRYSTAL experience. J. Clin. Oncol. 2008; 26: ASCO annual meeting, suppl; abstr 2

4) Bokemeyer C., et al. *KRAS* status and efficacy of first-line treatment of patients with metastatic colorectal cancer (mCRC) with FOLFOX with or without cetuximab: The OPUS experience. J. Clin. Oncol. 2008; 26: ASCO annual meeting, suppl; abstr 4000

5) Van Krieken J.H., et al. *KRAS* mutation testing for predicting response to anti-EGFR　therapy for colorectal carcinoma: proposal for an European　quality assurance program. Virchows Arch. 2008; 453: 417-431.

1. De Roock W., et al. Effects of *KRAS*, *BRAF*, *NRAS*, and PIK3CA mutations on the efficacy of cetuximab plus chemotherapy in chemotherapy-refractory metastatic colorectal cancer: a retrospective consortium analysis. Lancet Oncol. 2010; 11: 753-762.
2. Bivona TG., et al. FAS and NF-κB signaling modulate dependence of lung cancers on mutant EGFR. Nature. 2011; 471: 523-526.
3. Pharmaceutical and Medical Devices Safety Information No.219, November 2005. Ministry of Health, Labour and Welfare Pharmaceutical and Food Safety Bureau
4. Nelson MR., et al. Genome-wide approaches to identify pharmacogenetic contributions to adverse drug reactions. Pharmacogenomics J. 2008; 9: 23-33.
5. Dally AK. Genome-wide association studies in pharmacogenetics. Nature Reviews Genetics. 2010; 11: 241-246
6. Tanaka Y., et al. Genome-wide association of IL28B with response to pegylated interferon-α and ribavirin therapy for chronic hepatitis C. Nat. Genet. 2009; 41:　1105-1109.
7. Totoki Y., et al. High-resolution characterization of a hepatocellular carcinoma genome. Nat. Genet. 2011; 43: 464-469.
8. Adam J Bass, et al. Genomic sequencing of colorectal adenocarcinomas identifies a recurrent VTI1A-TCF7L2 fusion. Nat. Genet. 2011; 43: 964-968.
9. Cancer Genome Atlas Research Network. Integrated genomic analyses of ovarian carcinoma. Nature. 2011; 474: 609-615.
10. Agrawal N., et al. Exome sequencing of head and neck squamous cell carcinoma reveals inactivating mutations in NOTCH1. Science. 2011; 333: 1154-1157.
11. Stransky N., et al. The mutational landscape of head and neck squamous cell carcinoma. Science. 2011; 333: 1157-1160.
12. Blank PR., et al. *KRAS* and *BRAF* Mutation Analysis in Metastatic Colorectal Cancer: A Cost-effectiveness Analysis from a Swiss Perspective. Clin. Canc. Res. 2011; 17: 6338-6346.
13. Fleiss. JL., Measuring nominal scale agreement among many raters. Psych. Bull. 1971; 76: 378-382.
14. Storey JD., Tibshirani R. Statistical significance for genomewide studies. Proc. Natl. Acad. Sci.USA. 2003; 100: 9440-9445.
